# Using phosphoglucose isomerase-deficient (*pgi1*Δ) *Saccharomyces cerevisiae* to map the impact of sugar phosphate levels on d-glucose and d-xylose sensing

**DOI:** 10.1186/s12934-022-01978-z

**Published:** 2022-12-01

**Authors:** Celina Borgström, Viktor C. Persson, Oksana Rogova, Karen O. Osiro, Ester Lundberg, Peter Spégel, Marie Gorwa-Grauslund

**Affiliations:** 1 Division of Applied Microbiology, Department of Chemistry, Lund University, Lund, Sweden; 2 Centre for Analysis and Synthesis, Department of Chemistry, Lund University, Lund, Sweden; 3 Present Address: BioZone Centre for Applied Bioscience and Bioengineering, Department of Chemical Engineering and Applied Chemistry, University of Toronto, Toronto, Canada; 4 Present Address: Genetics and Biotechnology Laboratory, Embrapa Agroenergy, Brasília, DF 70770-901 Brazil

**Keywords:** *Saccharomyces cerevisiae*, *PGI1*, Metabolomics, Sugar sensing, d-xylose, d-fructose-6-phosphate, d-fructose-1,6-bisphosphate, PKA, Lignocellulose

## Abstract

**Background:**

Despite decades of engineering efforts, recombinant *Saccharomyces cerevisiae* are still less efficient at converting d-xylose sugar to ethanol compared to the preferred sugar d-glucose. Using GFP-based biosensors reporting for the three main sugar sensing routes, we recently demonstrated that the sensing response to high concentrations of d-xylose is similar to the response seen on low concentrations of d-glucose. The formation of glycolytic intermediates was hypothesized to be a potential cause of this sensing response. In order to investigate this, glycolysis was disrupted via the deletion of the phosphoglucose isomerase gene (*PGI1*) while intracellular sugar phosphate levels were monitored using a targeted metabolomic approach. Furthermore, the sugar sensing of the *PGI1* deletants was compared to the *PGI1*-wildtype strains in the presence of various types and combinations of sugars.

**Results:**

Metabolomic analysis revealed systemic changes in intracellular sugar phosphate levels after deletion of *PGI1*, with the expected accumulation of intermediates upstream of the Pgi1p reaction on d-glucose and downstream intermediates on d-xylose. Moreover, the analysis revealed a preferential formation of d-fructose-6-phosphate from d-xylose, as opposed to the accumulation of d-fructose-1,6-bisphosphate that is normally observed when *PGI1* deletants are incubated on d-fructose. This may indicate a role of *PFK27* in d-xylose sensing and utilization. Overall, the sensing response was different for the *PGI1* deletants, and responses to sugars that enter the glycolysis upstream of Pgi1p (d-glucose and d-galactose) were more affected than the response to those entering downstream of the reaction (d-fructose and d-xylose). Furthermore, the simultaneous exposure to sugars that entered upstream and downstream of Pgi1p (d-glucose with d-fructose, or d-glucose with d-xylose) resulted in apparent synergetic activation and deactivation of the Snf3p/Rgt2p and cAMP/PKA pathways, respectively.

**Conclusions:**

Overall, the sensing assays indicated that the previously observed d-xylose response stems from the formation of downstream metabolic intermediates. Furthermore, our results indicate that the metabolic node around Pgi1p and the level of d-fructose-6-phosphate could represent attractive engineering targets for improved d-xylose utilization.

**Supplementary Information:**

The online version contains supplementary material available at 10.1186/s12934-022-01978-z.

## Background

**Fig. 1:**
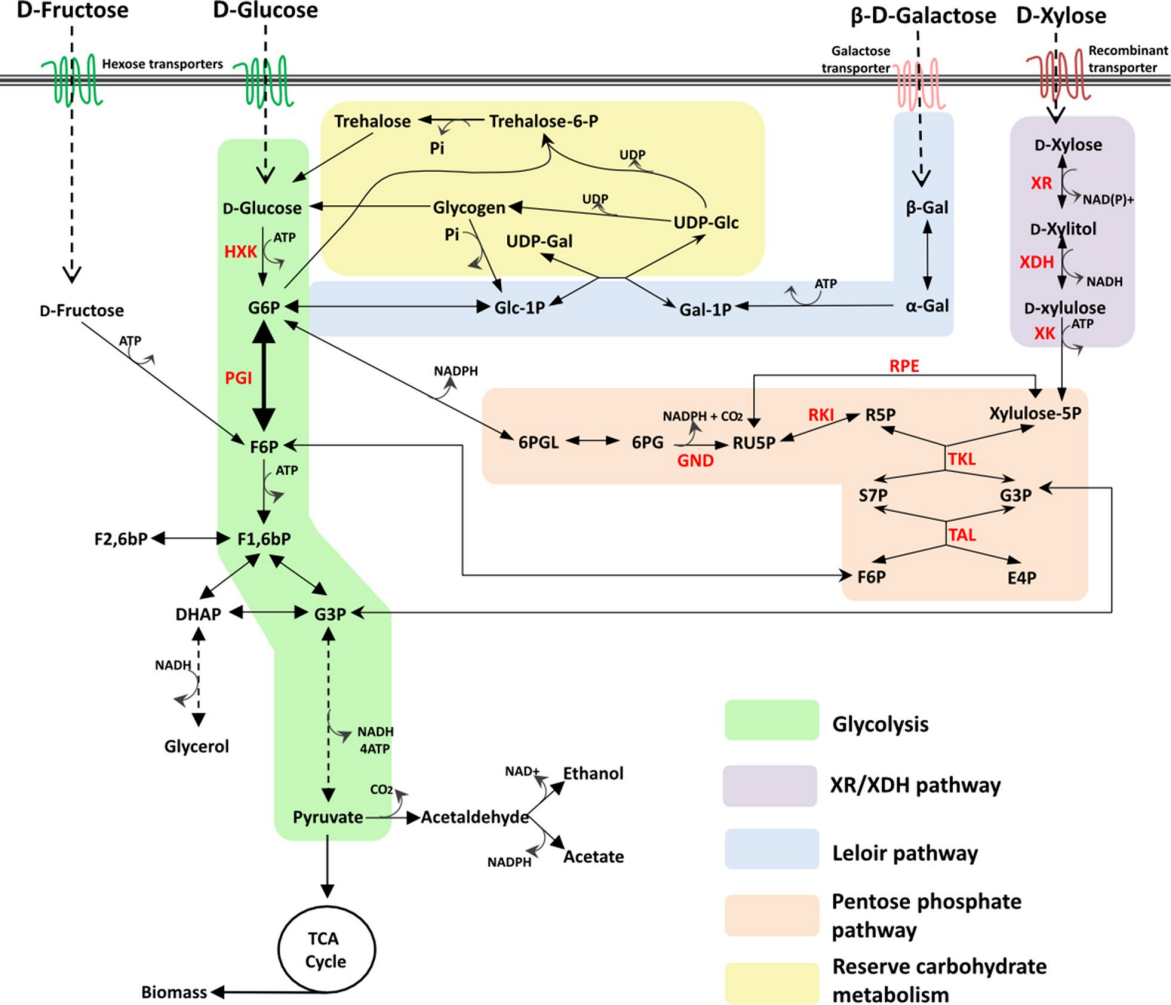
Metabolic map of S. cerevisiae, including d-xylose assimilation through the d-xylose reductase/xylitol dehydrogenase pathway. Glc: d-glucose; Gal: d-galactose; G6P: d-glucose-6-phosphate; F6P: d-fructose-6-phosphate; F1,6bP: d-fructose-1,6-bisphosphate; F2,6bP: d-fructose-2,6-bisphosphate; DHAP: dihydroxyacetone phosphate; G3P: glyceraldehyde-3-phosphate; 3PG: 3-phosphoglycerate; TCA cycle: tricarboxylic acid cycle; G1P: d-glucose-1-phosphate; Gal-1P: d-galactose-1-phosphate; UDP: uridine diphosphate; T6P: trehalose-6-phosphate; 6PGL: 6-phosphogluconolactone; 6PG: 6-phosphogluconate; RU5P: ribulose-5-phosphate; R5P: ribose-5-phosphate; S7P: sedoheptulose-7-phosphate; E4P: erythrose-4-phosphate; NADH: nicotinamide adenine dinucleotide; NADPH: nicotinamide adenine dinucleotide phosphate; ATP: adenosine triphosphate; HXK: hexokinase; PGI: phosphoglucose isomerase; XR: d-xylose reductase; XDH: xylitol dehydrogenase; XK: xylulokinase; RPE: ribulose-5-phosphate 3-epimerase; RKI: ribose-5-phosphate keto-isomerase; TKL: transketolase; TAL: transaldolase; UDP: uridine diphosphate

With the increasing demand for renewable and environmentally friendly products, biorefineries are attracting increased attention. In biorefineries, microorganisms can be used to convert waste streams into value-added fuels and fine chemicals such as bioethanol and polyhydroxyalkanoates (PHAs) [1]. Baker’s yeast, *Saccharomyces cerevisiae*, is a popular choice for these bioconversion processes since this GRAS eukaryote has high tolerance towards inhibitors found in, for instance, the hydrolysates of agricultural and forestry waste streams [2, 3]. However, since it cannot utilize d-xylose, a pentose which constitutes up to 30% of sugars in wood-based materials, *S. cerevisiae* has been genetically engineered by introducing either the d-xylose isomerase (XI) or the d-xylose reductase/xylitol dehydrogenase (XR/XDH) pathways [4–6]. Together with overexpression of genes encoding xylulokinase (XK) and enzymes from the non-oxidative pentose phosphate pathway (PPP), this enables a substantial flux of d-xylose-derived substrates to enter glycolysis at the level of d-fructose-6-phosphate (F6P) and glyceraldehyde-3-phosphate (G3P) (Fig. 1) [5–7].

Although ethanol production from d-xylose has nearly reached the maximum theoretical yield, the rate of d-xylose consumption is still significantly lower than that of d-glucose [8, 9]. Furthermore, co-utilization of d-xylose and d-glucose is not yet possible as d-glucose is preferentially consumed prior to d-xylose. Competition for transport into the cell may partly explain the sequential utilization of the sugars [10]; however, strains expressing engineered transporters with increased affinity for d-xylose are still not able to achieve sufficient co-consumption [11–13]. Since d-xylose is not a natural carbon source for *S. cerevisiae*, all genes of the engineered pathways are highly expressed even in the presence of d-glucose [14]. Therefore, d-xylose consumption is not expected to be directly affected by the catabolite repression that normally controls the sugar preference of *S. cerevisiae* by altering the gene expression. Catabolite repression in *S. cerevisiae* is tightly regulated by the level of d-glucose and by the presence or absence of other naturally used carbon sources, such as d-galactose [15]. The absence of d-glucose, and the presence of certain sugars such as d-galactose or d-fructose, results in the expression of genes which enable the catabolism of these alternative sugars [16]. It remains unclear whether or not, and by extension how, d-xylose is sensed by *S. cerevisiae*.

Using a previously established biosensor system responsive to variations in the three main d-glucose signaling routes (*HXT1*p for the Snf3p/Rgt2p pathway; *SUC2*p for the SNF1/Mig1p pathway; *TPS1*p for the cAMP/PKA pathway) [17], we recently showed that d-xylose assimilation product(s) are sensed in recombinant yeast carrying the XR/XDH pathway, something that does not occur in wild-type *S. cerevisiae* [18]. However, the observed sugar signaling response to high concentrations of d-xylose resembled the one observed on low concentrations of d-glucose [18]. This gave rise to the hypothesis that during the incorporation of carbon from d-xylose into glycolysis, one or more metabolic intermediates with sugar signaling functionalities are formed. d-Glucose-6-phosphate (G6P), trehalose-6-phosphate (T6P), and d-fructose-1,6-bisphosphate (F1,6bP) have been proposed as the major sugar signaling intermediates for d-glucose catabolism [15, 19–22]. On d-xylose, *S. cerevisiae* can produce ethanol via F1,6bP but also glycogen via G6P [23], indicating that both G6P and F1,6bP intermediates are generated (Fig. 1). The formation of these intermediates from d-xylose may be triggering the observed sugar sensing response. G6P production from d-xylose requires phosphoglucose isomerase (Pgi1p) activity, encoded by the *PGI1* gene (YBR196C), to interconvert G6P and F6P. Consequently, the deletion of *PGI1* is hypothesized to alter the sugar sensing response of these d-xylose utilizing strains.

In the present study, *PGI1* gene was deleted in recombinant d-xylose-utilizing XR/XDH strains to alter the levels of glycolytic intermediates. The intermediate levels were further manipulated by changing the type and level of carbon sources added to the incubation medium. Alterations in sugar metabolism and changes in sugar signaling were examined with the previously developed fluorescent biosensor system combined with targeted profiling of intracellular sugar phosphates, with the objective to identify putative connections between sensing and the measured intermediates.

## Results and discussion

### Growth on monomeric sugars

In order to manipulate the intracellular level of glycolytic intermediates, and G6P in particular, *PGI1* gene was deleted in three recombinant d-xylose-fermenting strains carrying fluorescent sugar signaling biosensors. The strains carried either the *HXT1*p-GFP biosensor (TMB3752), the *SUC2*p-GFP biosensor (TMB3755) or the *TPS1*p-GFP (TMB3757) biosensor, reporting on the Snf3p/Rgt2p, the SNF1/Mig1p, and the cAMP/PKA sugar signaling pathways, respectively. Details on strain construction and validation can be found in Additional file 1: S1.

**Fig. 2:**
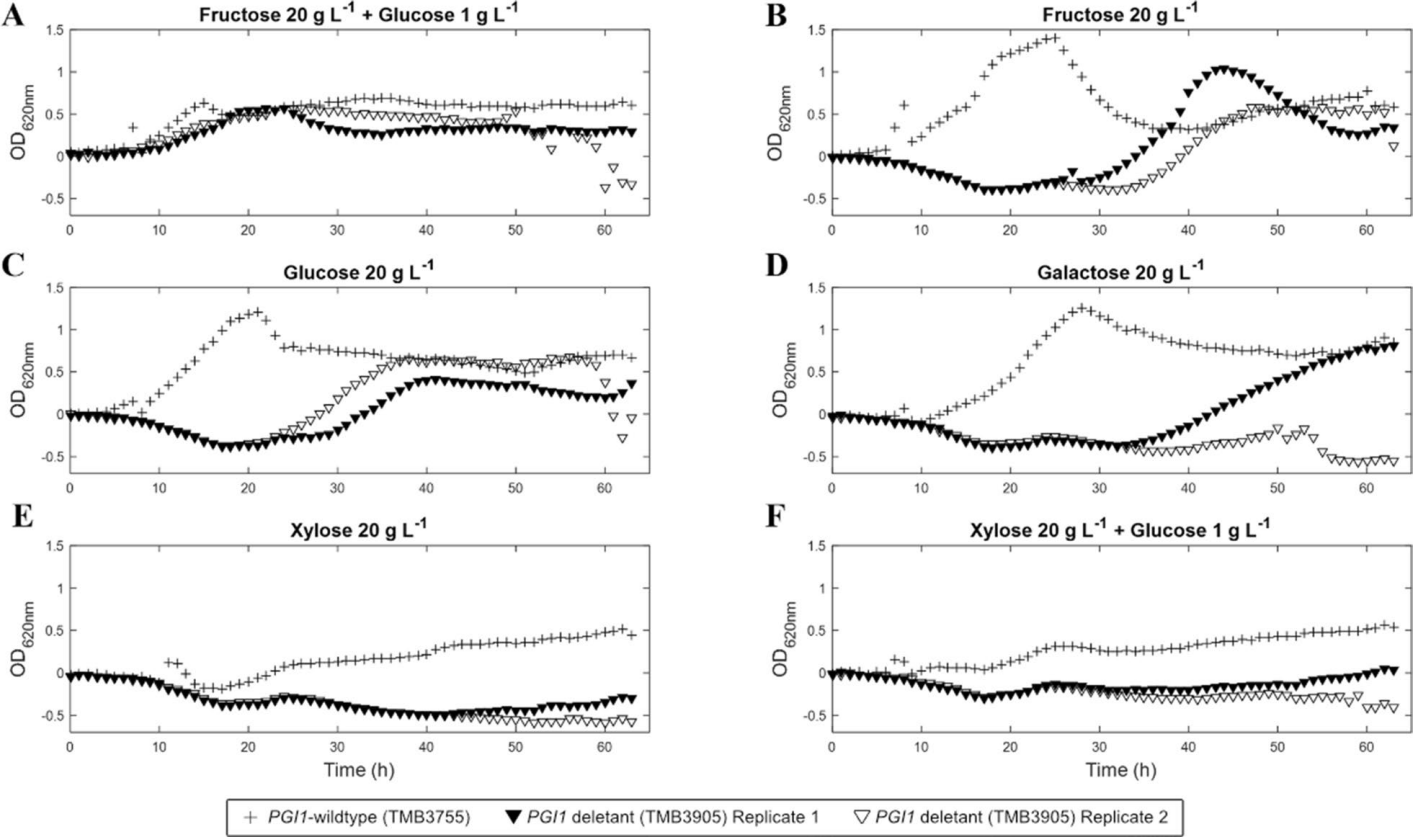
Growth of *PGI1*-wildtype and deletants during microscale cultivations in defined medium. Two replicates for the deletant strain (TMB3905, open and closed triangles) and one replicate for the *PGI1*-wildtype strain (TMB3755, plusses) are shown for the *SUC2*p biosensor

*Saccharomyces cerevisiae* strains with inactive Pgi1p are known to lose their ability to grow on single monomeric sugars such as d-glucose and d-fructose [24, 25]. Abolishment of growth on d-glucose has been attributed to three potential factors: (1) the imbalance caused when ATP is consumed during initial glucose phosphorylation but not regenerated further down in glycolysis [26], (2) an accumulation of toxic levels of G6P [26], and (3) the inability to channel carbon into the lower glycolysis for precursor production and NADPH reoxidation as the flux through the PPP is considered too low [20, 25, 27]. Similarly, growth on d-fructose as sole carbon source has not yet been reported, likely because G6P is needed for anabolic processes and can only be generated by gluconeogenesis via Pgi1p (Fig. 1). Indeed, *PGI1* deletants have been reported to grow on d-fructose supplemented with a small amount (1 g L^−1^) of d-glucose [28]. In order to screen the growth response of the *PGI1* deletants to a larger number of carbon source combinations, including d-xylose, micro-scale cultivations were carried out in 96-well microplates (Fig. 2).

**Fig. 3:**
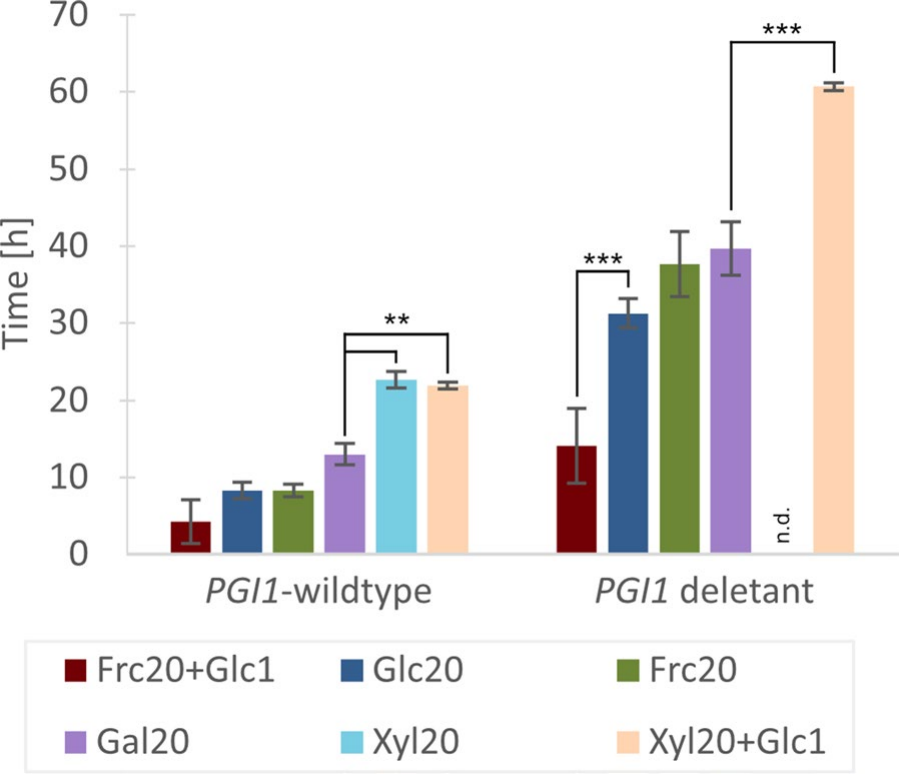
Lag phase duration (h) for microscale cultivations in defined media. Measurements in microtiter plates from two to four replicates for each strain in each carbon source. Replicates that did not initiate growth at all were omitted. Frc20: 20 g L^−1^ d-fructose; Glc1: 1 g L^−1^ d-glucose; Glc20: 20 g L^−1^ d-glucose; Gal20: 20 g L^−1^ d-galactose; Xyl20: 20 g L^−1^ d-xylose. n.d. = not determined, *p < 0.05, **p < 0.01, ***p < 0.001

Cultivation in microplates is typically limited by incomplete aeration, medium evaporation, and non-linear correlations between OD_620_ and biomass. Our experiments were performed in quadruplicates and the aforementioned limitations did not appear to have interfered with the determination of lag phase duration and growth in microtiter plates (Fig. 3). The longer lag phase seen for the *PGI1* deletants in aerobic shake flasks containing rich YPFG medium (Additional file 1: Fig. S2) was also observed in the microscale cultures in defined YNB-FG medium, albeit with larger variations (Fig. 2A). In the *PGI1*-wildtype strains, exponential growth on single fermentable sugars (d-fructose, d-glucose and d-galactose) started almost immediately (Figs. 2B–D, 3). Growth was also recorded on d-xylose for the *PGI1*-wildtype strains (engineered with the XR/XDH pathway), albeit with a longer lag phase (22.7 h) compared to other monomeric sugars (8.3–13 h) and with a linear growth pattern (Figs. 2E, 3).

Overall, the *PGI1* deletants displayed a significant increase in lag phase duration compared to the *PGI1*-wildtype strains. Surprisingly, and in contrast to previous reports, the *PGI1* deletants were able to grow on monomeric d-glucose, d-galactose and d-fructose, albeit with prolonged lag phases compared to *PGI1*-wildtype strains. Additionally, an increased heterogeneity between biological replicates was observed (Figs. 2B–E, 3), as not all biological replicates of the *PGI1* deletants initiated growth. A possible explanation for the growth of *PGI1* deletants on monomeric sugars may lie in the genetic makeup of the *PGI1*-wildtype strains: the strains have been engineered to ferment d-xylose by introduction of heterologous XR and XDH genes, overexpression of a d-xylose-selective mutated Gal2p transporter [29], and by overexpression of the PPP genes *TKL1* and *TAL1* [30]. The latter modification is likely of importance for the growth on monomeric d-glucose since the PPP flux is thought to be one of the limiting factors in *PGI1* deletants [28]. In fact, a doubling of the activities of 6-phosphogluconate dehydrogenase (GND), ribulose-5-phosphate-3-epimerase (RPE) and transaldolase (TAL) (Fig. 1) has been shown to be one of the mechanisms used in suppressor mutants of *PGI1* deletant strains [31]. Additionally, *PGI1* deletants of the yeast *Kluyveromyces lactis* were able to grow on d-glucose only, but lost this ability upon additional disruptions in the PPP [32]. Similarly, the overexpressed PPP might explain the growth seen in d-galactose media. d-Galactose enters the yeast central metabolism at the level of G6P through isomerization, phosphorylation, UDP-transfer and phosphate transfer (Fig. 1). This assimilation requires UDP-d-glucose, which has been shown to accumulate in *PGI1* deletants pre-grown on YPFG [33]. In conjunction with higher flux through the PPP, this may also explain the growth observed on sole d-galactose in the *PGI1* deletants.

In contrast, upregulation of PPP genes cannot explain growth on d-fructose in the d-xylose-engineered strains of the present study, since G6P cannot be generated under these conditions. In a previous study, Corominas and colleagues observed that during early exponential growth on YPFG media, *PGI1* deletants accumulated G6P, UDP-d-glucose and the storage carbon glycogen [33]. Since the pre-cultures of the present study all consist of rich YPFG medium, it is possible that the cells being inoculated into d-fructose medium had sufficient storage of glycogen to be broken down into d-glucose-6-phosphate (via d-glucose and d-glucose-1-phosphate; Fig. 1) to support growth on d-fructose. The growth phase of the pre-culture may be crucial as glycogen was shown to decrease fourfold between early and late exponential phase [33], which might offer an explanation, together with the benefit of the upregulated PPP, to why other studies also employing YPFG as pre-culture did not observe growth on d-fructose for their *PGI1* deletants.

In the cultivations containing d-xylose as a single carbon source, no growth was recorded for the *PGI1* deletants (Fig. 2E), whereas the addition of 1 g L^−1^ d-glucose enabled some growth in two of the replicates, but with a lag phase of over 60 h (Fig. 2F). d-Xylose is expected to enter glycolysis at the F6P and G3P nodes (Fig. 1), implying *PGI1* deletants could theoretically grow on d-xylose through the same mechanisms as on d-fructose. However, the lower carbon flux and limited glycogen production might exceed the maintenance needs and prevent growth on d-xylose.

### Targeted metabolite profiling reveals systemic changes in intracellular sugar phosphate levels after *PGI1* deletion

To further study the phenotype of *PGI1* deletants assimilating various carbon sources, concentrations of intracellular sugar phosphates were determined in the *PGI1*-wildtype and *PGI1* deletion strains carrying each biosensor. This analysis focused on sugar phosphates from the glycolysis since several of these have been implicated in the regulation of sugar metabolism *S. cerevisiae* [19, 22]. The metabolite profiling also included other sugar phosphates with possibly unknown regulatory roles from closely related pathways such as the pentose phosphate pathway, the Leloir pathway, and the trehalose pathway.

**Fig. 4:**
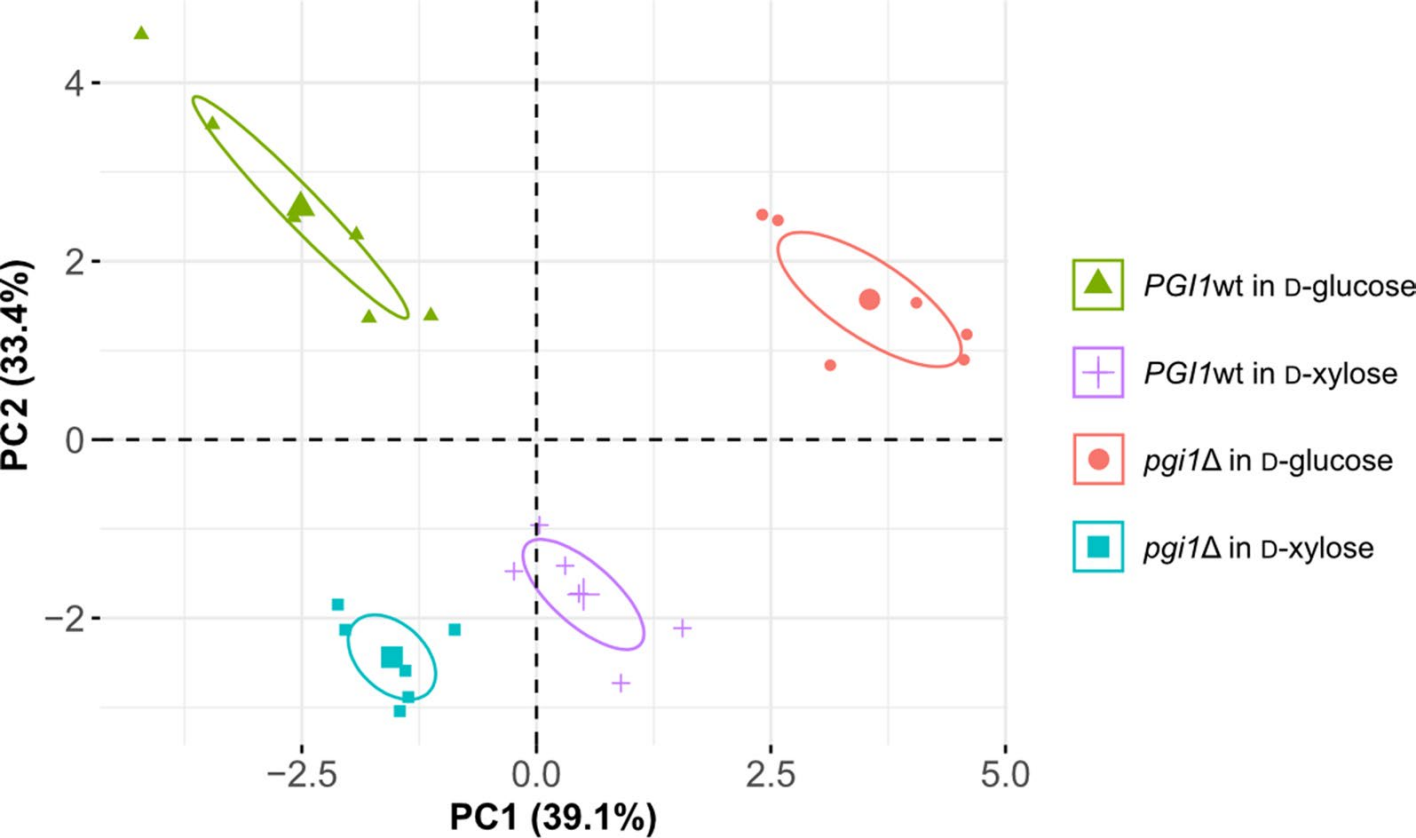
Score scatter plot obtained from a principal component analysis of intracellular sugar phosphate levels. Each data point represents one replicate of a strain and condition. Proximity between points represent similarities in the overall levels of measured intracellular sugar phosphates. First (PC1) and second (PC2) principal components are shown, explaining 72.5% of the variation in the data. Data points are grouped by strain (*PGI1*wt: *PGI1*-wildtype strains, *pgi1*∆: *PGI1* deletant strains) and incubation condition (30 min in 20 g L^−1^ d-glucose or d-xylose): Ellipses illustrate 95% confidence intervals for each group. Score scatter plots for additional components can be found in Additional file 1: Fig. S3

First, a principal component analysis (PCA) was conducted to generate an overview of alterations in metabolite levels associated with the *PGI1* deletion and variation in the carbon source. This analysis revealed systematic changes in intracellular sugar phosphate accumulation in the *PGI1*-wildtype and deletant strains on the two different carbon sources. The first and second principal components (PCs), PC1 and PC2, accounted for 39.1% and 33.4% of the variation in the data, respectively (Fig. 4; Scree plot is shown in Additional file 1: Fig. S3). The score scatter plot for the two first PCs revealed a clear impact of both the sugar used during the incubation (d-glucose vs. d-xylose) and the genotype of the strain (*PGI1*-wildtype vs. *PGI1* deletants) on levels of sugar phosphates (Fig. 4).

**Fig. 5:**
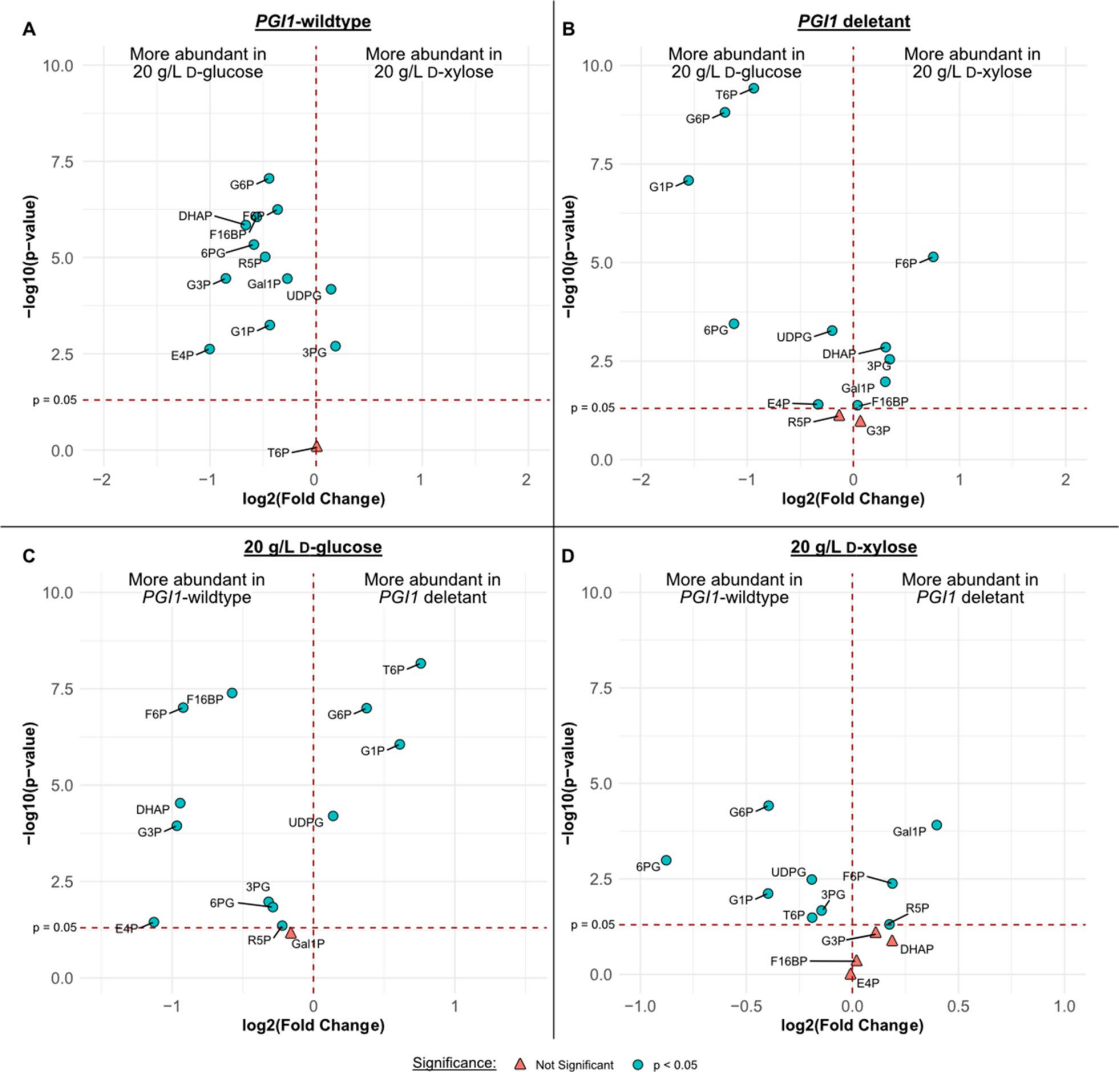
Comparison of relative intracellular sugar phosphate concentrations between subgroups. **A**, **B** The effect of the carbon source on strains; positive values on the x-axis (log2 fold-change) implies higher intracellular sugar phosphate concentrations when incubated on 20 g L^−1^ d-xylose, while negative values indicate higher concentrations when incubated on 20 g L^−1^ d-glucose for the *PGI1*-wildtype strains (**A**) or the *PGI1* deletants (**B**). **C**, **D** The effect of *PGI1* deletion on each carbon source; positive values on the x-axis (log2 fold-change) implies higher intracellular sugar phosphate concentrations in the deletion strain, while negative values indicate higher abundance in the *PGI1*-wildtype strain when incubated on 20 g L^−1^ d-glucose (**C**) or 20 g L^−1^ d-xylose (**D**). Significant (p < 0.05) changes are marked with blue circles, insignificant (p > 0.05) changes are marked with red triangles. Absolute values for the concentrations of intracellular sugar phosphates can be found in Fig. S4. G6P: d-glucose-6-phosphate; F6P: d-fructose-6-phosphate; F1,6bP: d-fructose-1,6-bisphosphate; DHAP: dihydroxyacetone phosphate; G3P: glyceraldehyde-3-phosphate; 3PG: 3-phosphoglycerate; G1P: d-glucose-1-phosphate; Gal1P: d-galactose-1-phosphate; UDPG: uridine diphosphate d-glucose/galactose; T6P: trehalose-6-phosphate; 6PG: 6-phosphogluconate; R5P: ribose-5-phosphate; S7P: sedoheptulose-7-phosphate; E4P: erythrose-4-phosphate

Next, specific alterations in intracellular metabolite levels were investigated. In general, the *PGI1*-wildtype strains showed lower levels of intermediates when grown on d-xylose compared to d-glucose (Fig. 5A), as expected from the lower flux generally observed through the d-xylose pathway compared to the glycolysis. For the *PGI1* deletant strains, a media-dependent effect on metabolite levels was observed, with higher levels of metabolites upstream of Pgi1p on d-glucose (G6P; T6P; G1P, d-glucose-1-phosphate) and higher levels of metabolites downstream of Pgi1p on d-xylose (F6P; S7P, sedoheptulose-7-phosphate; DHAP, dihydroxyacetone phosphate) (Fig. 5B).

In 20 g L^−1^ d-glucose, *PGI1* deletants showed a hyper-accumulation of G6P and accumulation of intermediates formed from G6P through glycogen and trehalose synthesis pathways (G1P and T6P, respectively) (Fig. 5C). Elevated levels of 6-phosphogluconate (6PG) and erythrose-4-phosphate (E4P) were detected in the d-glucose media compared to the d-xylose media (Fig. 5B). Formation of these two intermediates (6PG and E4P) indicates that the PPP is active, since they cannot form via the inactivated Pgi1p, which supports the proposed mechanism mentioned earlier whereby the increased expression of PPP in our engineered strains is enabling the unprecedented growth on monomeric sugars for *PGI1* deletants. The deletion strains also showed decreased concentrations of intermediates downstream of Pgi1p (F1,6bP; F6P; DHAP; G3P; 3PG, 3-phosphoglycerate) which could be expected as a direct consequence of the *PGI1* deletion (Fig. 5C). Similarly, a decrease in 6PG, as well as downstream intermediates such as S7P and E4P, was also observed, confirming that the oxidative PPP flux is limited in G6P-accumulating *PGI1* deletants [26, 27]. Although the data showed that concentrations of intermediates downstream of the Pgi1p reaction were lower in the deletant strains compared to the *PGI1*-wildtype strains, the compounds were not fully depleted (Additional file 1: Fig. S4).

In both the *PGI1*-wildtype and deletion strains, d-xylose was converted via the XR/XDH pathway, followed by the non-oxidative PPP after which the carbon entered the lower glycolysis at the F6P and G3P nodes (Fig. 1). Consequently, *PGI1*-wildtype strains were expected to form upstream intermediates (G6P, T6P, G1P, 6PG) from F6P via Pgi1p when incubated on d-xylose, while deletant strains were not. Indeed, formation of these upstream sugar phosphates was observed on d-xylose for the *PGI1*-wildtype strain but not for the deletants (Fig. 5D). Accumulations of F6P and S7P were recorded in the deletants on d-xylose, which could result from the overall decreased glycolytic flux leading to accumulation of intermediates and the ability of the *PGI1*-wildtype strain to convert F6P into intermediates upstream of the Pgi1p reaction such as G6P. Curiously, d-galactose-1-phosphate (Gal1P) was observed to accumulate in the *PGI1* deletant strains. Given the decrease in G6P levels, deletant strains would be expected to also consume Gal1P via the Leloir pathway as the flux is directed to G6P formation via G1P (Fig. 1). The persistence of Gal1P in the deletant strains might be explained by a lack of UDP-d-glucose, which is required for its integration into the glycolysis. Previous studies have reported the accumulation of glycogen in *PGI1* deletants [33], which consumes UDP-d-glucose and thus may partly explain the Gal1P accumulation seen on d-xylose. Unfortunately, it was not possible to determine the UDP-d-glucose levels in this experiment as it could not be distinguished from UDP-d-galactose.

### Sugar signaling responses and further indications of d-xylose not being recognized as fermentable

**Fig. 6:**
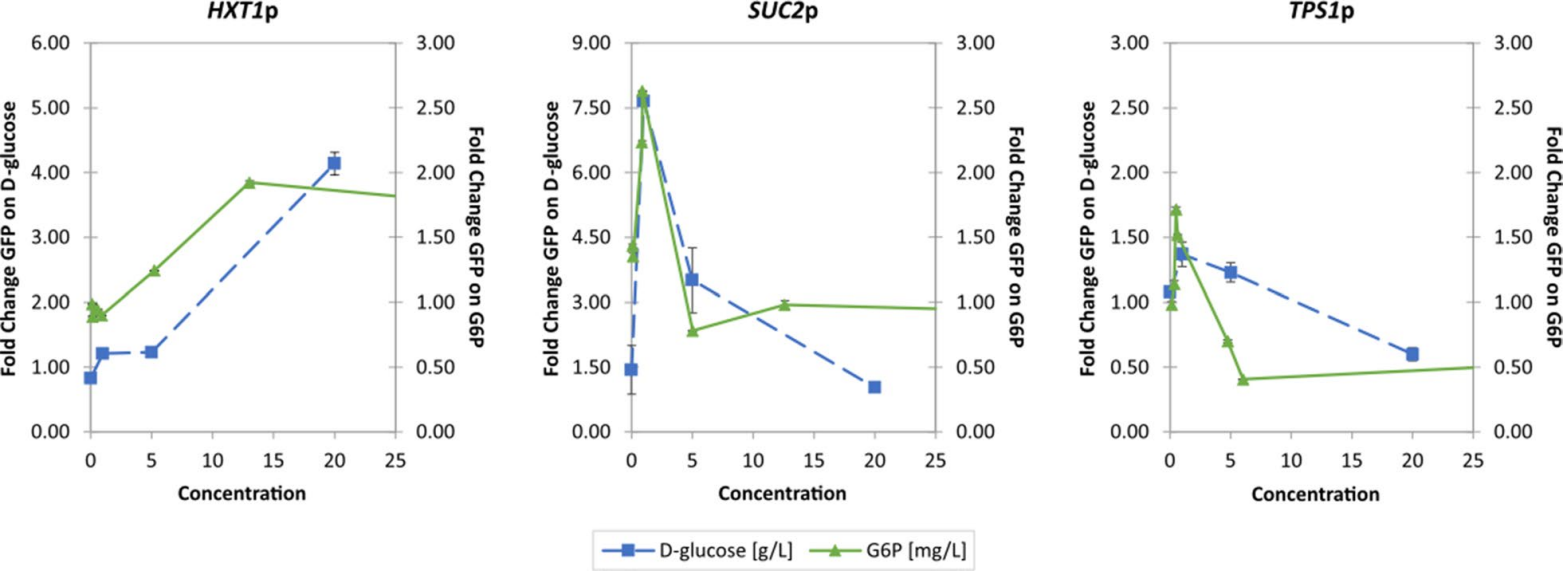
Similarities in sugar sensing responses in the three biosensors. GFP fold change is normalized to the fluorescence at 0 h after incubation on YPFG. Green lines with triangle markers show the biosensor responses for different concentrations of intracellular d-glucose-6-phosphate levels [mg L^−1^] modulated by *PGI1* deletion and variation of media composition. Blue dashed lines with square markers show the biosensor responses to various concentrations of extracellular d-glucose [g L^−1^] (adapted from Brink et al. 2016 [17]). d-glucose-6-phosphate graphs are cropped for clarity; uncropped graphs can be found in Additional file 1: Fig. S5

The yeast sugar signaling response was explored in the *PGI1*-wildtype and *PGI1* deletant strains by recording the activity of biosensors previously constructed to report on the three main sugar signaling routes [17, 18]. We first attempted to plot the biosensor responses directly as a function of the intracellular sugar phosphate levels, using combined data from both strains and carbon sources. The biosensor responses to d-glucose were found to have similarities to the biosensor responses for G6P (Fig. 6). However, correlations between biosensor responses and other intracellular sugar phosphates proved difficult to explain (Additional file 1: Fig. S5), likely due to the time separation between metabolite sampling (30 min of incubation) and optimal GFP expression (6 h).

**Fig. 7:**
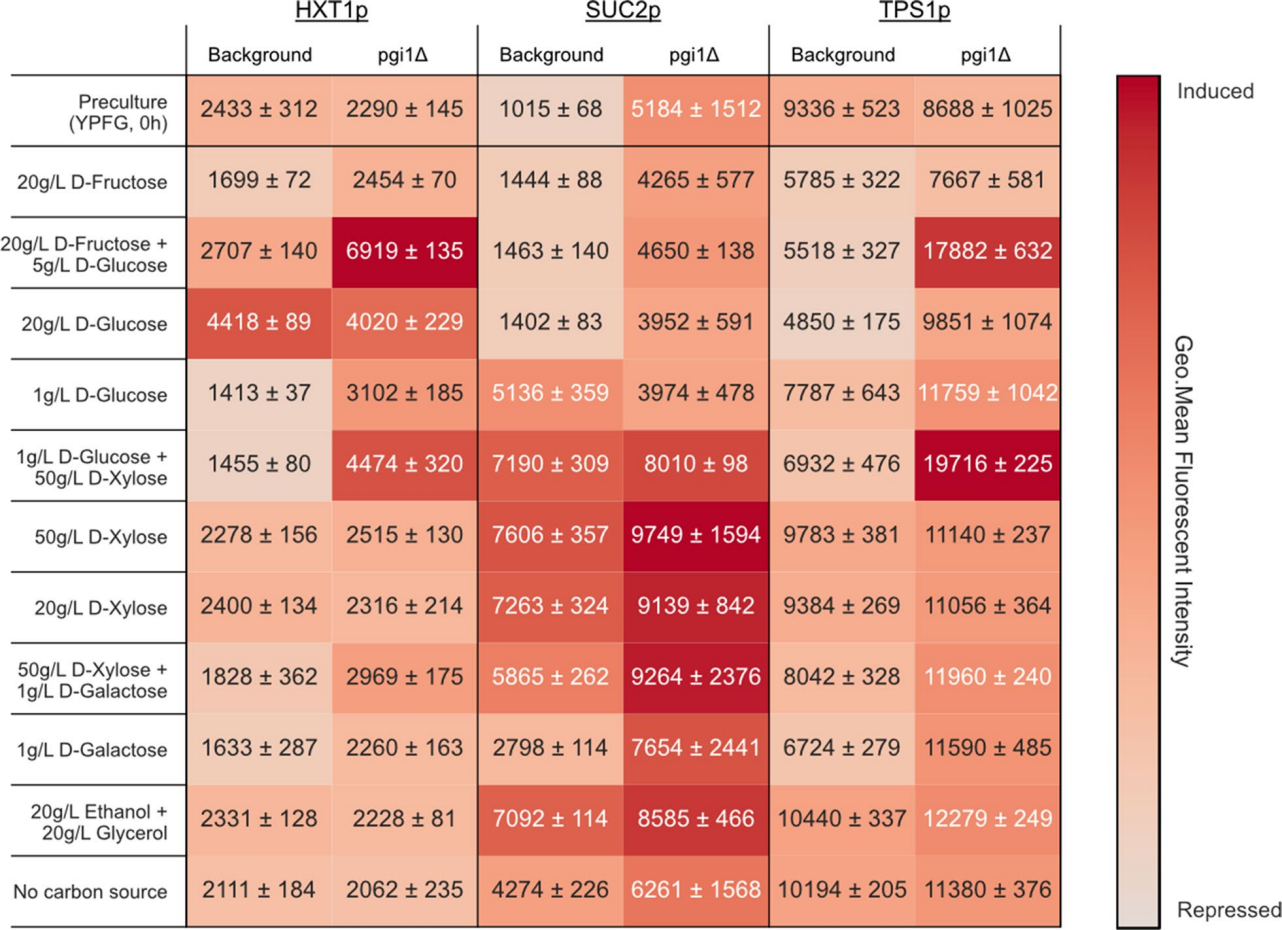
Promoter responses to different carbon sources in *PGI1*-wildtype and *PGI1* deletant strains. The first row shows the fluorescent intensities at 0 h after the YPFG pre-culture, and subsequent rows show the fluorescent intensities after 6 h of incubation in different YNB-sugar media for the *PGI1*-wildtype strains and for the *PGI1* deletants. The heatmap hue is adjusted to each individual sensor for clarity since each biosensor inherently differs in maximum fluorescent intensity

To further understand the sugar signaling responses, the strains were instead evaluated in d-glucose, d-xylose, d-fructose, and d-galactose at various concentrations, similar to a previous experiment performed by Osiro et al. (2018) [18]. The strains were incubated in the sugar concentrations previously evaluated for growth (20 g L^−1^) as well as in concentrations that have been used in previous sugar sensing studies (50 g L^−1^ and 1 g L^−1^) [17, 18, 34]. No significant differences in fluorescent intensities were recorded between 20 and 50 g L^−1^ d-xylose for any of the strains and conditions (see Fig. 7), indicating that 20 g L^−1^ was sufficient to elicit a similar signaling response as the one previously observed at 50 g L^−1^ [18]. Notably, since 50 g L^−1^ d-xylose elicits signals resembling those on low levels of d-glucose [18], 1 g L^−1^ d-glucose and 1 g L^−1^ d-galactose were also included as conditions in this study for comparison. Although we made attempts to repress the sensors prior to the signaling assays (to produce fold change values rather than fluorescent intensities), repression of the *SUC2*p and *TPS1*p sensors was not achievable due to the d-glucose toxicity seen in *PGI1* deletants. Consequently, all strains were pre-incubated on a mixture of 20 g L^−1^ d-fructose with 1 g L^−1^ d-glucose prior to inoculation into the various conditions. Although minor subpopulations were observed in some fluorescence histograms for the *PGI1*-wildtype strains (Additional file 1: Fig. S6), they did not alter the interpretation of the results and were included in the average fluorescent intensities shown in Fig. 7.

In the *PGI1*-wildtype strains, the expected biosensor responses were recorded, i.e. *HXT1*p induction at high d-glucose concentrations (20 g L^−1^) as well as repression at low-to-no d-glucose; full induction of *SUC2*p at low concentrations of d-glucose (1 g L^−1^), with repression at higher levels (> 5 g L^−1^) and only basal induction in carbon-free media; and *TPS1*p repression in response to preferred carbon sources such as d-glucose, d-fructose, and d-galactose (Fig. 7) [16]. Additionally, we observed the previously reported induction of *SUC2*p on d-xylose [18] as well as *TPS1*p induction, indicating both a carbon starvation response and a decrease in PKA activity, which further supports the notion that d-xylose is not recognized as a fermentable carbon source [35–38]. The combination of 20 g L^−1^ ethanol with 20 g L^−1^ glycerol resulted in a biosensor expression pattern that was also strikingly similar to the one observed on 20 g L^−1^ d-xylose (repressed *HXT1*p, induced *SUC2*p, and induced *TPS1*p). In both conditions, the assimilated carbon sources enter glycolysis downstream of the Pgi1p reaction (F6P and G3P for d-xylose, DHAP and PEP for glycerol and ethanol), which could indicate a connection between the level of, or flux through, some of these downstream intermediates and the observed response. Although in opposition of this, the condition containing 20 g L^−1^ d-fructose, which also enters glycolysis downstream of the Pgi1p reaction, instead showed a *SUC2*p and *TPS1*p response that was closer to that seen on 20 g L^−1^ d-glucose rather than d-xylose. Notably, the low *HXT1*p response seen on 20 g L^−1^ d-fructose aligned with previous data which indicated weaker PKA-induced phosphorylation of Rgt1p on d-fructose than on d-glucose [39].

### Deletion of *PGI1* alters the sugar signaling response

The *PGI1* deletion resulted in changes to the biosensor response for nearly all conditions (Fig. 7). Induction of the *HXT1*p reporter was maintained at high d-glucose level, but it was now observed at low d-glucose levels as well, which points towards a role of G6P and/or T6P on *HXT1*p induction. Even higher induction of *HXT1*p was observed in the combinations of low d-glucose with d-fructose or low d-glucose with d-xylose, whereas neither high d-fructose nor high d-xylose alone led to induction. One possible explanation is that a certain combination of intermediates (either at certain levels or at certain rates of formation) upstream (G6P/T6P) and downstream (F6P/F1,6bP) of the Pgi1p reaction is necessary to achieve this induction.

The *TPS1*p biosensor remained repressed by d-fructose in the *PGI1* deletants but a weaker repression was observed on both d-glucose and d-galactose. *TPS1*p expression is repressed by PKA activity [16, 40] and consequently the persistence of repression in the d-fructose condition likely reflects an active PKA. The cAMP/PKA pathway has been hypothesized to be controlled directly by intracellular metabolite concentrations, rates of formation, and metabolic fluxes in addition to the established impact of the extracellular Gpr1p sensor and the intracellular Ras1/2p components [16, 41]. For instance, PKA has been shown to be activated by F1,6bP [19], which is formed from d-fructose via F6P but cannot be formed in *PGI1* deletants on d-glucose or d-galactose. The formation of F1,6bP could explain why *TPS1*p remains repressed when supplied with d-fructose, but not when given d-glucose or d-galactose. Interestingly, the *TPS1*p and *HXT1*p biosensors both show the highest increase in fluorescence in response to the mix of 1 g L^−1^ d-glucose with 50 g L^−1^ d-xylose and to the mix of 5 g L^−1^ d-glucose with 20 g L^−1^ d-fructose in the *PGI1* deletants. The simultaneous induction of both these sensors at once is quite unexpected since the full induction of *HXT1*p is dependent on hyperphosphorylation of the Rgt1p transcription factor by active PKA; hence full *HXT1*p induction is expected only when *TPS1*p is repressed by PKA activity [16, 40, 42].

The *SUC2*p biosensor showed an overall increase in fluorescence in the *PGI1* deletant compared to the *PGI1*-wildtype strain, with the exception of the 1 g L^−1^ d-glucose condition where a lower signal was observed. Compared to the condition lacking a carbon source, the *PGI1* deletants still showed repression in response to high concentrations of d-glucose (20 g L^−1^), and now also displayed the same repression in response to low concentrations of the sugar (1 g L^−1^) likely due to the accumulation of G6P. These findings are in line with the proposed role of G6P as a key regulator of glucose repression: G6P acts via the SNF1/Mig1p pathway, likely by dephosphorylating SNF1 through the Reg1p-Glc7p phosphatase via an unknown signaling mechanism, which ultimately leads to the repression of *SUC2*p and other genes [16, 22, 43, 44]. As such, the increased concentration of intracellular G6P seen for *PGI1* deletants in response to d-glucose is expected to lead to *SUC2*p repression. Counterintuitively, this was not observed in growth conditions including d-galactose, however this may be due to the lower concentration (1 g L^−1^) and the less rapid accumulation of G6P via the Leloir pathway [45]. The *SUC2*p repression was relieved on d-xylose and ethanol-glycerol media, further confirming that d-xylose is not sensed as a repressing fermentable sugar. Perhaps even more interestingly, repression was also relieved in the d-glucose/d-xylose medium (1 g L^−1^ + 50 g L^−1^) and maintained in the d-fructose (20 g L^−1^) medium. This hints towards a role of metabolites downstream of G6P on catabolite repression, as G6P is not expected to form during the utilization of d-fructose nor d-xylose in the *PGI1* deletant.

The cause of the *TPS1*p induction on mixtures of d-glucose with either d-xylose or d-fructose is not known, but it might indicate that *TPS1*p has become partially deregulated from PKA activity in *PGI1* deletants. This would also be in line with the results showing the curious co-induction of *HXT1*p and *TPS1*p mentioned earlier. It has been hypothesized that an increase in trehalose synthesis, and consequently *TPS1*p expression, might act as a way for the cell to free up inorganic phosphates (bound in the form of G6P) in these deletants [46]. However, given that d-glucose and d-galactose are both expected to result in G6P accumulation in the *PGI1* deletant, this does not explain why *TPS1*p is induced in the mixed 50 g L^−1^ d-xylose media containing 1 g L^−1^ d-glucose, but not in the mixed media containing 1 g L^−1^ d-galactose. Possibly, the elevated *TPS1*p induction seen on d-glucose relies partly on the activation of the extracellular Gpr1p d-glucose sensor and partly on the intracellular G6P levels. Alternatively, there may be differences in the flux rates and final metabolites formed when incubated in d-glucose compared to d-galactose (for instance the G6P formation rate may be too low on d-galactose). Additional experiments investigating the signaling response and changes in metabolite levels while varying concentrations of sugars that enter upstream and downstream of Pgi1p might shed more light on the causes of this peculiar signaling state.

### The putative role of d-fructose-bisphosphate regulation on d-xylose utilization

**Fig. 8:**
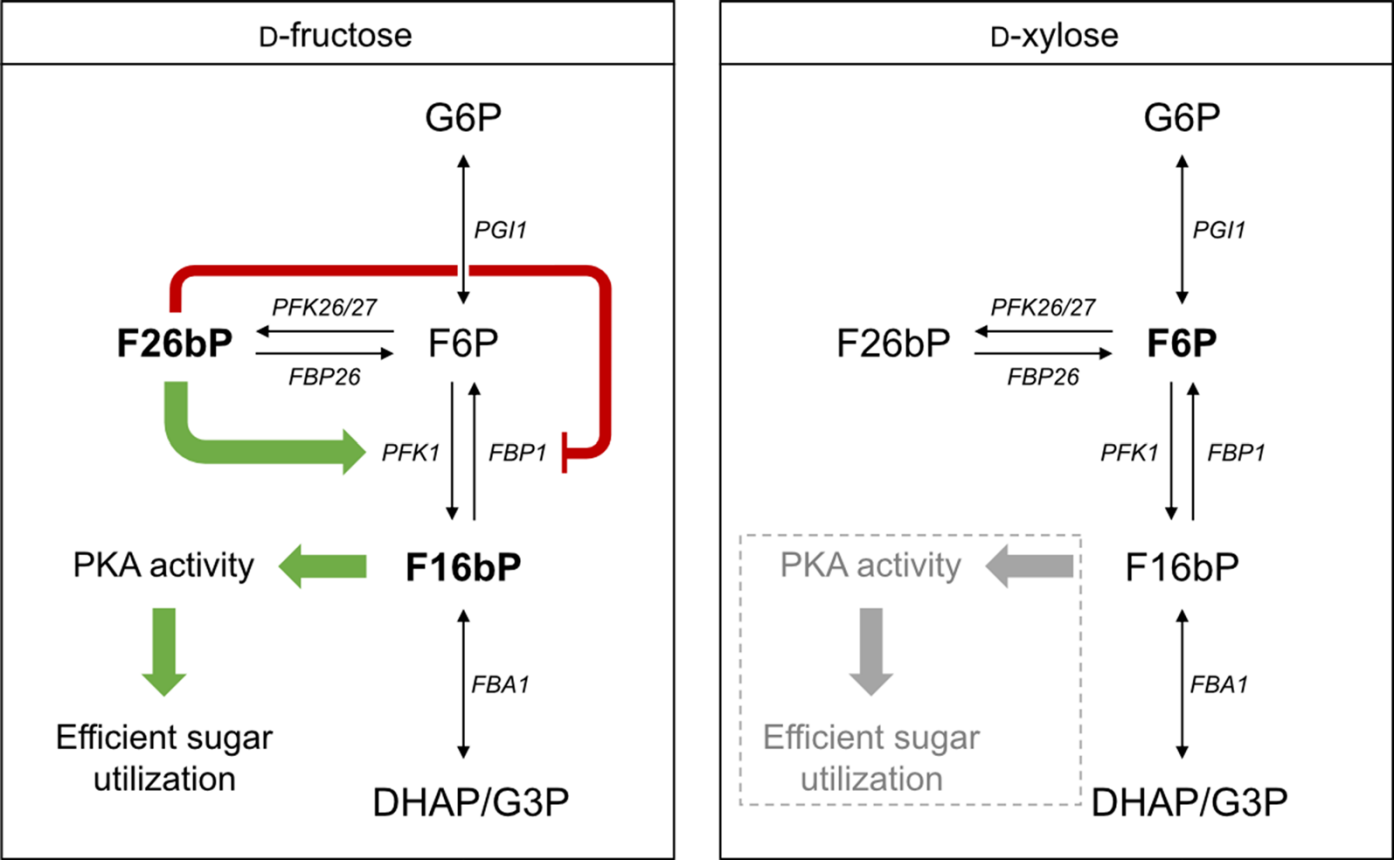
Putative d-fructose bisphosphate regulation mechanism in relation to d-xylose utilization. Lack of *PFK27* expression in response to d-xylose, but not in response to d-fructose, may be linked with low utilization rates and lack of PKA activation. Accumulated sugar phosphates are marked in bold font [43]. Green arrows indicate positive regulation, red hammerheads indicate negative regulation

The *PGI1* deletants have previously been reported to accumulate F1,6bP from d-fructose [43]. However, in the metabolite profiling of the present study (Fig. 5D) accumulation of F6P from d-xylose was instead observed. Hence, it is possible that the differences in sugar signaling seen between d-xylose and d-fructose may be linked to d-fructose phosphate levels or formation rates (in addition to the repressive effect of G6P). F1,6bP is synthesized from F6P by phosphofructo-1-kinase (Pfk1p) and can be converted back to F6P by d-fructose-1,6-bisphosphatase (Fbp1p) [47]. The activities of both Pfk1p and Fbp1p are allosterically regulated by d-fructose-2,6-bisphosphate (F2,6bP), a metabolite synthesized by Pfk26/27p, which activates Pfk1p and inactivates Fbp1p [47–49]. It has been shown that the *PFK27* gene is induced by d-fructose but not by d-xylose [49], which could lead to differences in F2,6bP levels in these two conditions. In extension, the decreased levels of F2,6bP might cause the decrease in F1,6bP levels on d-xylose via Pfk1p/Fbp1p regulation. Interestingly, increased levels of F1,6bP have been shown to enhance PKA activity [19]. Thus, the variation in F1,6bP levels might also affect the regulation of PKA differently between the two conditions (Fig. 8). This is of particular interest, since low PKA activity has been pointed out as a possible component that results in poor d-xylose utilization [16, 34, 37, 50]. Additionally, activation of PKA by F1,6bP in the d-fructose condition may lead to the inactivation of the SNF1 kinase and thus the observed *SUC2*p repression. Conversely, the lack of F1,6bP may lead to the induction of *SUC2*p seen on d-xylose, and consequently the expression of suboptimal catabolic genes which further impedes strain performance. To investigate the potential role of F2,6bP on poor d-xylose utilization, one possibility would be to deregulate *PFK26/27* genes in d-xylose-utilizing strains and measure both the F2,6bP levels and the sugar signaling over time on different carbon sources. An increased *PFK26/27* expression on d-xylose would be expected to result in a similar sugar signaling state as seen on d-fructose, and potentially also lead to an improved performance on d-xylose. Indeed, in a previous study by Shen and colleagues, *PFK27* overexpression was found to be one of the changes that arose during adaptive laboratory evolution for improved d-xylose utilization [51]. However, reintroduction of *PFK27* overexpression in the parental strain did not show improved growth in the studied conditions and strain [51], and further testing of the potential benefits of *PFK27* has yet to be performed. Future experiments exploring this topic could investigate the hypothesized decrease of F1,6bP levels in d-fructose media after *PFK27* deletion in *PGI1* deletants, and the potential increase of F1,6bP levels in d-xylose media upon *PFK27* overexpression. Additionally, since we hypothesize that the cAMP/PKA pathway will show increased activity upon overexpression of *PFK27*, the sugar signaling and d-xylose utilization of *PGI1*-wildtype strains carrying this mutation should be examined as well.

## Conclusions

By applying metabolite profiling on strains carrying fluorescent sugar signaling reporters, systemic changes in intracellular sugar phosphate levels were mapped upon deletion of the *PGI1* gene. In d-glucose media, *PGI1* deletants showed the expected accumulation of sugar phosphates upstream of the Pgi1p reaction and depletion of downstream intermediates. In d-xylose media, the deletion strains instead accumulated sugar phosphates downstream of Pgi1p while metabolites upstream were depleted.

Rather than accumulating F1,6bP—which has been reported to accumulate om *PGI1* deletants cultivated in d-fructose media—the *PGI1* deletants instead showed accumulation F6P in the d-xylose medium. We hypothesize that the decreased level of F1,6bP on d-xylose is a result of unfavorable *PFK26/27* regulation. Decreased levels of F1,6bP are expected to result in decreased PKA activity which may result in suboptimal utilization of d-xylose. Consequently, overexpression of *PFK27* should be explored as a potential way of increasing F1,6bP formation, which may in extension improve d-xylose utilization.

The choice of carbon source was also shown to have a different influence on the signaling pathways in the *PGI1* deletants than in the *PGI1*-wildtype strains. In particular, responses to sugars entering the metabolism above the Pgi1p reaction (d-glucose and d-galactose) changed the most. Peculiarly, the simultaneous exposure to sugars both upstream and downstream of the Pgi1p reaction caused opposite responses for the Snf3p/Rgt2p and cAMP/PKA pathways. Future experiments could provide more concrete connection between sugar signaling and intracellular sugar phosphates by performing targeted metabolite profiling on cultures incubated on a wider variety of carbon sources. Overall, the biosensor responses seen in this study confirmed the importance of several glycolytic intermediates on sugar sensing.

## Materials and methods

### Strains, plasmids, and media

The plasmids and yeast strains used in the present study are presented in Tables 1 and 2, respectively. Primers used for amplification and confirmation of DNA fragment integration are found in Additional file 1: Table S1. *S. cerevisiae* strains were grown at 30 °C on solid or liquid YP (10 g L^−1^ yeast extract; 20 g L^−1^ peptone) or YNB (6.7 g L^−1^ yeast nitrogen base without amino acids; 50 mM potassium hydrogen phthalate buffer, pH 5.5) with or without 15 g L^−1^ agar and supplemented with suitable sugars. YP supplemented with 20 g L^−1^ d-fructose and 1 g L^−1^ d-glucose (YPFG) was routinely used for the *PGI1* deletants. Geneticin (G418; 200 mg L^−1^) was supplemented to the medium to maintain the episomal plasmid pCfB2312 and aureobasidin A (AbA; 0.3 mg L^−1^) was supplemented to the medium for selection of uptake of the pUG62AUR-F12.Pgi1 plasmid. *Escherichia coli* strain NEB5α (New England Biolabs, Ipswich, MA, USA) was used for preparation and amplification of plasmids and was grown in liquid or solid Lysogeny Broth (LB) medium (10 g L^−1^ tryptone, 5 g L^−1^ yeast extract, 5 g L^−1^ NaCl; with or without 15 g L^−1^ agar) supplemented with 100 mg L^−1^ ampicillin, at 37 °C.

**Table 1:** Plasmids used in the present study

Plasmid	Relevant genotype	Reference
pUG62AUR	LoxP–AbAR–LoxP;	[52]
pUG62AUR-F12.Pgi1	PGI1(US)-pUG62AUR-PGI1(DS)	This study

US: region upstream of PGI1; DS: region downstream of PGI1

**Table 2:** *S. cerevisiae* strains used in the present study

Strain	Biosensor	Relevant genotype	References
TMB3700	None	W303-1A (MATa, *trp1-1 leu2-3112 his3-11 ade2-1 ura3-1 can1-100*); *TRP1, HIS3*, *ura3*::M3499 (*ADE2*)	[17]
TMB3752	HXT1p	TMB3700; *can1*::*HXT1p-yEGFP3-tPGK1*, *SPB1/PBN1::YIp128GAL2mut*; VAC17/MRC1::TKL-TAL*; Chr X-2/XI-5/XII-4::XR-XDH-XK**	[18]
TMB3755	SUC2p	TMB3700; *can1*::*SUC2p-yEGFP3-tPGK1*, pCfB2312, *SPB1/PBN1::YIp128GAL2mut*; VAC17/MRC1::TKL-TAL*; Chr X-2/XI-5/XII-4::XR-XDH-XK**	[18]
TMB3757	TPS1p	TMB3700; *can1*::*TPS1p-yEGFP3-tPGK1*, pCfB2312, *SPB1/PBN1::YIp128GAL2mut*; VAC17/MRC1::TKL-TAL*; Chr X-2/XI-5/XII-4::XR-XDH-XK**	[18]
TMB3902	HXT1p	TMB3752; *PGI1::AbAR*	This study
TMB3905	SUC2p	TMB3755; *PGI1::AbAR*	This study
TMB3907	TPS1p	TMB3757; *PGI1::AbAR*	This study

^*^TKL-TAL = *pFBA1-TKL1-tPDC1, pTPI1-TAL1-tCPS1*

^**^XR-XDH-XK = *pTDH3-SpXYL1.2-tADH1, pTEF1-SsXDH-tGPM1, pPGI1-XKS1-tPYK1*

### Genetic methods

*Saccharomyces cerevisiae* DNA sequences were amplified with PCR using either Phusion High-Fidelity DNA polymerase or DreamTaq polymerase, from Thermo Fisher Scientific (Waltham, MA, USA). Primers (Additional file 1: Table S1) were ordered from Eurofins Genomics (Ebersberg, Germany). PCR products were purified with GeneJET PCR Purification Kit and plasmids were purified with GeneJET Plasmid Miniprep Kit (Thermo Fisher Scientific). DNA concentrations were measured with BioDrop Duo spectrophotometer at 280 nm (BioDrop, Cambridge, UK).

Genomic yeast DNA was extracted using glass beads as previously described [53]. Restriction enzymes and T4 DNA ligase (5 U µL^−1^) were purchased from Thermo Fisher Scientific. Digestions and DNA amplifications were verified with gel electrophoresis using 0.8% (w/v) agarose. DNA extractions from agarose gels were made with GeneJET Gel Extraction Kit (Thermo Fisher Scientific).

Competent *E. coli* cells were prepared and transformed with the method described by Inoue and colleagues (1990). Competent *S. cerevisiae* cells were prepared and transformed with the lithium acetate method described by Gietz and Schiestl [54], with an addition of 10% (v/v) DMSO prior to the heat shock [55]. Transformations and gene integration sites were verified using diagnostic colony PCR.

### Construction of plasmids and *PGI1* deletants

Two sequences corresponding to 500 bp upstream and 500 bp downstream of *PGI1* were amplified from genomic yeast DNA isolated from strain TMB3752, using primers 1–4 (Additional file 1: Table S1). The two fragments were ligated at the *Nhe*I site introduced by the forward primer of flank 1 and the reverse primer of flank 2, producing fragment F12 that was subsequently PCR amplified and inserted into the backbone vector pUG62AUR using restriction enzymes AvrII/SalI, producing the pUG62AUR-F12.Pgi1 plasmid.

*PGI1* deletants were constructed by replacing the endogenous *PGI1* gene with the linearized plasmid containing the AbA resistance marker gene (*AbAR*) through homologous recombination by transforming *Nhe*I linearized pUG62AUR-F12.Pgi1 into strains TMB3752, TMB3755 and TMB3757. After transformation, the strains were incubated in liquid YPFG medium for 2 h before selective plating. Transformants were selected on YPFG agar plates containing AbA and G418. Colonies were re-streaked on solid media with YPFG and YP supplemented with 20 g L^−1^ d-glucose (YPD). Colonies that were able to grow on YPFG but not on YPD were selected and used in colony PCR to verify the *PGI1* deletion using primers 5 and 6 (Additional file 1: Table S1).

### Enzymatic assays

Single colonies were grown in 5 mL YPFG medium in 50 mL conical tubes overnight. Cells were then washed, resuspended and incubated in YPD for 2 h before harvesting and protein extraction using Y-PER (Thermo Fisher Scientific, Waltham, MA, USA) according to the supplier’s instructions. Total protein concentrations were determined using the Coomassie (Bradford) Protein Assay Kit (Thermo Fisher Scientific) microplate procedure according to the supplier’s instructions, using bovine serum albumin as standard. Phosphoglucose isomerase activity was determined in technical and biological duplicates, using the method of Maitra and Lobo [56].

### Shake flasks cultures

Single colonies were inoculated into 5 mL YPFG in 50 mL conical tubes and incubated in 30 °C and 180 rpm overnight. Pre-cultures were then used to inoculate 25 mL YPFG in 250 mL baffled shake flasks to an optical density at 620 nm (OD_620_) of approximately 0.1. Cultures were carried out in biological duplicates, 30 °C and 180 rpm. Optical density was monitored using a Ultrospec 2100 Pro spectrophotometer (Amersham Biosciences, Chicago, IL, USA).

### Microtiter plate cultures

Pre-cultures were prepared by inoculation of single colonies into 5 mL YPFG medium in 50 mL conical tubes and incubation in 30 °C and 180 rpm overnight, after which they were used to inoculate 96-well microtiter plates to an OD_620_ of approximately 0.1 in YNB medium supplemented with various carbon sources. OD_620_ was measured with an automated spectrophotometer (Multiskan Ascent, Thermo Electron Corporation, Waltham, MA, USA) every 10 min for 64 h. Growth was evaluated in biological duplicates. To confirm that an increase in absorbance was a result of growth and not the microtiter plate drying out or an artefact of the instrumentation, all wells were manually checked for cell pellets at the end of the cultivations. Negative controls, consisting of YNB supplemented with 20 g L^−1^ d-fructose and 1 g L^−1^ d-glucose (YNB-FG) inoculated with sterile water only were also used to verify that no growth was obtained. Maximal growth rates (µmax) were identified as the highest linear slope found when the natural logarithms of the optical densities were plotted against time whereas lag phase duration was determined as the time point where maximal growth rate was initiated. Maximal growth rates and lag phase durations were statistically analyzed using two-factor analysis of variance (ANOVA) and the Tukey Honest Significant Difference post-hoc test to find significant changes.

### Fluorescence measurements

Fluorescence intensity (FI) was analyzed at the single-cell level using a BD Accuri C6 flow cytometer connected to a BD CSampler autosampler (Becton–Dickinson, Franklin Lakes, NJ, USA) with an excitation wavelength of 488 nm, detection of GFP using a 533/30 bandpass filter (FL1-H channel), and detection of propidium iodide stain using a 670 lowpass filter (FL3-H channel). For each sample 20,000 events were recorded with a threshold of 80,000 for the FSC-H channel. FlowJo v10.7.1 software (Treestar, Inc., San Carlos, CA, USA) was used to analyze the data from each biological replicate. Permeabilized cells were stained using propidium iodide (1.32 µg/mL) and were gated away using the FL3-H channel prior to analysis of mean geometrical GFP fluorescent intensity in the FL1-H channel. At least 10,000 events remained for the GFP analysis from each sample after the gating was complete.

For fluorescence measurements in microtiter plates with various sugars, pre-cultivation was performed from a single colony inoculation into a 50 mL conical tube containing 5 mL of YPFG medium which was incubated overnight at 30 °C and 180 rpm. The pre-culture was used to inoculate a microtiter plate containing YNB supplemented with various sugars to an initial OD_620_ of 0.5 and incubation was performed at 30 °C, 800 rpm, for 6 h. For fluorescence intensities associated with metabolomic measurements, flow cytometry samples were taken from the shake flasks at time points 0 h, 0.5 h and 6 h.

### Culture conditions for metabolomics experiments

Single colonies were inoculated into 25 mL of YPFG in 250 mL baffled shake flasks and incubated at 30 °C at 180 rpm until late exponential phase. Cells were then inoculated into 50 mL of YNB supplemented with 20 g L^−1^ of either d-glucose or d-xylose in 250 mL baffled shake flasks at an initial OD_620_ of 0.5. Cultures were incubated at 30 °C and 180 rpm for 30 min before sampling for metabolite profiling.

### Sample storage, preparation and derivatization

Quenching of metabolism and sampling for sugar metabolite profiling were performed as described by Bergdahl, Heer [57]. Briefly, 10 mL cell culture was added to 40 mL − 40 °C methanol (60% in water). Samples were incubated for 5 min at − 40 °C before centrifugation at − 9 °C and 3220*g* for 5 min. The supernatant was discarded, and the cell pellet stored at − 80 °C.

Intracellular metabolites were extracted using mechanical disruption in methanol [58]. The cells were resuspended in 80% methanol (pre-cooled to − 80 °C) and bead beaten with a Precellys 24 homogenizer (Bertin Instruments, Montigny-le-Bretonneux, France) using the Precellys lysing kit with the following program: 3 cycles of 30 s of agitation at 6500 rpm with 30 s rest in between. The Cryolys cooling unit (Bertin Instruments, Montigny-le-Bretonneux, France) was used with liquid nitrogen to cool the samples during bead beating. Cell debris and beads were removed by centrifugation at 21,130*g* and 4 °C for 5 min.

Prior to metabolite profiling, all samples were centrifuged (4 °C, 14,000 rpm, 10 min). A quality control (QC) stock sample was prepared by mixing aliquots of each sample. From each sample and the QC stock 200 µL was placed into 1.5 mL Eppendorf tubes and 70 μL of chloroform was added to yield a chloroform/alcohol ratio of 3/7 (v/v). Subsequently, samples were vortexed for 5 s and placed in a freezer (− 20 °C) for 2 h. Then, 10 µL of 20 µg mL^−1^ 2-deoxy-d-glucose 6-phosphate (internal standard, IS) was added and samples were extracted twice with cold water (8 °C; 2 × 200 μL). The aqueous layers were pooled and evaporated for approximately 3 h until dry in a modular miVac concentrator (Genevac Ltd., Ipswich, United Kingdom).

The derivatization consisted of two steps. First, 20 μL of methoxyamine hydrochloride in dry pyridine (MOX; Thermo Fisher Scientific) was added to the dried samples, after which the samples were mixed by vortexing and left at room temperature overnight. Then, 6 μL of 1-methylimidazol and 12 μL of propionic acid anhydride were added and the samples were again mixed by vortexing. Finally, the derivatization reaction was allowed to continue for 30 min at 37 °C, after which the samples were evaporated for approximately 3 h until dry under a flow of nitrogen gas (Pierce Reacti-Vap III; Thermo Fisher Scientific, Minneapolis, MN, USA). The samples were stored at − 20 °C. Prior to UHPLC-MS/MS analysis, 100 μL of water containing 0.1% formic acid (HCOOH) was added to the samples.

### UHPLC-MS/MS analysis of derivatized sugar phosphates

Derivatized sugar phosphates were analyzed on an Agilent 1290 Infinity UHPLC system coupled with an Agilent 6495 QqQ-MS (Agilent Technologies, Santa Clara, CA) operated in dynamic multiple-reaction-monitoring (MRM) mode, as previously described in detail [59]. Briefly, chromatographic separation was performed on a Waters Acquity HSS-T3 1.8 μm, 2.1 × 50 mm C18 column (Waters Corporation, Milford, MA, USA) with mobile phase A composed of water and mobile phase B of MeOH, both containing 2% of HCOOH. The gradient was: 5% B for 1 min, then changing linearly from 5 to 30% B from 1 to 3 min, then 30 to 40% B from 3 to 6 min, hold at 40% B from 6 to 10 min, then 40 to 70% B from 10 to 12.5 min, hold at 70% B from 12.5 to 15 min, and then 70 to 99% B from 15 to 17 min, hold at 99% B for 0.5 min, followed by re-equilibration to 5% B in 0.5 min and to 0.1% B in 2 min. The flow rate was 0.5 mL min^−1^, the column temperature was 40 °C, and the injection volume was 1 μL. The mass spectrometer was operated in negative electrospray ionization (ESI) mode with a gas temperature of 150 °C, a gas flow of 16.1 L min^−1^, a nebulizer pressure of 20 psi, a sheath gas temperature of 350 °C and flow-rate of 11 L min^−1^, a capillary voltage of 3000 V, a nozzle voltage of 1000 V, an iFunnel high pressure RF of 80 V, an iFunnel low pressure RF of 40 V, a fragmentor voltage of 380 V, and a cell acceleration voltage of 5 V. MRM transitions are given in Additional file 1: Table S2.

### Data processing and statistical analysis of UHPLC-MS/MS data

Data were processed using MassHunter Qualitative Analysis and Quantitative Analysis software (Agilent Technologies, Santa Clara, CA, USA). Pentose phosphates were partially co-eluting and therefore integrated as a sum, except for one of the ribose-5-phosphate tautomers which was clearly resolved from the others. The second, most intense, of the two tautomers for d-glucose 6-phosphate was selected for quantification. UDP-d-glucose and UDP-d-galactose co-eluted and are reported as a single signal. Statistical analysis was performed in R (version 3.6.0.). Method relative standard deviation (RSD) was determined from three independently extracted and derivatized QC samples analyzed in quadruplicates over 24 h. The repeatability for the entire method, including extraction and derivatization, was 14.28% (Additional file 1: Table S2). Peak areas were normalized to the area of the IS and log2-transformed to conform to normality. Principal component analysis (PCA) was conducted using prcomp (stats). Fold changes and significance levels for volcano plots were derived from linear models (lm, stats) and anova (Anova, car), and graphs were produced using ggplot (ggplot2).

## Supplementary Information

**Additional file 1: Table S1.** Primers used in the study. **Table S2.** Multiple-reaction-monitoring (MRM) transitions and retention times of LC–MS analysis of sugar phosphates **Fig. S1.** *PGI1* deletion confirmation in the biosensor strains. **Fig. S2.** Representative aerobic growth curves (OD_620_) of *PGI1*-deficient strain and *PGI1*-wildtype strain. **Fig. S3.** Top 10 principal components in metabolomics analysis explaining 99.4% of the variation observed. **Fig. S4.** Concentration of intracellular sugar phosphates in *PGI1*-wildtype and *PGI1* deletants on 20 g L^−1^ D-glucose or 20 g L^−1^ D-xylose. **Fig. S5.** Fluorescence intensity (FI) of biosensors as a function of intracellular sugar phosphate concentrations obtained from the *PGI1*-wildtype and deletion strains grown on D-glucose or D-xylose. **Fig. S6.** Histogram of biosensors *HXT1*p (**A**, **B**), *SUC2*p (**C**, **D**) and *TPS1*p (**E**, **F**) without (**A**, **C**, **E**) and with (**B**, **D**, **F**) *PGI1* gene deletion.

## Data Availability

All data generated in this study is included in the published article and Additional file 1, full datasets for flow cytometry and metabolic profiling are available from the corresponding author upon request.
