# Supplementary material for: Using phosphoglucose isomerase-deficient (pgi1Δ) Saccharomyces cerevisiae to map the impact of sugar phosphate levels on d-glucose and d-xylose sensing
Source: Microb Cell Fact. 2022 Dec 1;21:253. doi: 10.1186/s12934-022-01978-z (PMC9713995; doi:10.1186/s12934-022-01978-z)
Supplement: Supplementary file 1 — Additional file 1: Table S1. Primers used in the study. Table S2. Multiple-reaction-monitoring (MRM) transitions and retention times of LC–MS analysis of sugar phosphates Fig. S1. PGI1 deletion confirmation in the biosensor strains. Fig. S2. Representative aerobic growth curves (OD620) of PGI1-deficient strain and PGI1-wildtype strain. Fig. S3. Top 10 principal components in metabolomics analysis explaining 99.4% of the variation observed. Fig. S4. Concentration of intracellular sugar phosphates in PGI1-wildtype and PGI1 deletants on 20 g L−1 D-glucose or 20 g L−1 D-xylose. Fig. S5. Fluorescence intensity (FI) of biosensors as a function of intracellular sugar phosphate concentrations obtained from the PGI1-wildtype and deletion strains grown on D-glucose or D-xylose. Fig. S6. Histogram of biosensors HXT1p (A, B), SUC2p (C, D) and TPS1p (E, F) without (A, C, E) and with (B, D, F) PGI1 gene deletion. [file 12934_2022_1978_MOESM1_ESM.docx]

# Additional file for:

**Using phosphoglucose isomerase-deficient *Saccharomyces* *cerevisiae* to further understand the role of glycolytic intermediates in strains engineered for** d-**xylose utilization**

Celina Borgström^1^ & Viktor Persson^1^, Oksana Rogova^2^, Karen O. Osiro^1^, Ester Lundberg^1^,
Peter Spégel^2^, Marie F. Gorwa-Grauslund^1^

^1^ Division of Applied Microbiology, Department of Chemistry, Lund University, Lund, Sweden.

^2^ Centre for Analysis and Synthesis, Department of Chemistry, Lund University, Lund, Sweden

**S1 - Construction and validation of *PGI1* deletant strains**

*PGI1* was deleted through homologous recombination using flanks of 500 base pairs upstream and downstream of the *PGI1* open reading frame. The structural gene was replaced with AbAR, a gene variant of the native *AUR1* (YKL004W), conferring resistance to aureobasidin A, and transformants were selected on a medium containing the antibiotic. *PGI1* deletion was confirmed by diagnostic PCR using the primers in Table S1, and were further confirmed using enzymatic activity measurements (Fig. S1).

Table S1. Primers used in the study. Inserted restriction sites are underlined.

| **Primer number** | **Primer name** | **Restriction site** | **Sequence (5’-3’)** | **Purpose** |
| --- | --- | --- | --- | --- |
| 1 | F1_FP | *Nhe*I | AAGCTAGCATCGACCAGCATTAGTAGGGG | Amplification of flanking region upstream of *PGI1* promoter |
| 2 | F1_RP | *Avr*II | TACCTAGGATCACGTACTTCTCACCGTCAAG |  |
| 3 | F2_FP | *Sal*I | TAGTCGACATGAAAGATACTCGCACTGGAAG | Amplification of flanking region DS of *PGI1* terminator |
| 4 | F2_RP | *Nhe*I | AAGCTAGCATGTAGAGCCGTGTTTTGTTC |  |
| 5 | F1_US_FP | - | GAAATGTATGTTGCTGGCAC | Verification of *PGI1* deletion |
| 6 | F2_DS_RP | - | CAAGTTCCAGGCTAAAGAAG |  |

'

Specific activity (µmol min^-1^ mg^-1^)

Figure S1. PGI1 deletion confirmation in the biosensor strains. Pgi1p specific activity in the background strains (white) and the PGI1 deletants (grey). For the enzyme assay cultures were incubated with 20 g L^-1^ glucose for 2 h before harvesting, to ensure full expression of any available gene.

The specific activity of the background strains was approximately 0.04-0.09 µmol min^-1^ mg^-1^ whereas the specific activity of the *PGI1*-deleted strains was below 0.003 µmol min^-1^ mg^-1^ (Fig. S1). This indicated that the deletion of *PGI1* was successful, but it also confirmed that there was no other enzyme catalyzing the interconversion between G6P and F6P.

Growth abolishment of *PGI1* deletants on d-glucose was also confirmed on solid media using YP supplemented with 20 g L^-1^ d-glucose (YPD) (data not shown). In parallel, *PGI1* deletants were shown to grow on 20 g L^-1^ d-fructose supplemented with 1 g L^-1^ d-glucose medium (YPFG) as in previous studies using other strain backgrounds (Aguilera, 1986) (data not shown).

Aerobic shake flask cultures were performed on the constructed biosensors and their respective deletant strains in YPFG medium. The *PGI1* deletants exhibited a clear growth delay compared to the background strains (Fig. S2): whereas the background strains displayed a lag phase of three to five hours before entering exponential growth, the *PGI1* deletants had corresponding lag phases that lasted for around 18h. Also, the deletants showed 50 % reduced maximum specific growth rates as compared to the background strains (0.22-0.25 vs. 0.44-0.45 h^-1^, respectively).

Figure S2. Representative aerobic growth curves (OD_620_) of PGI1-deficient strain (open symbols) and background strain (closed symbols). Cells were cultivated in shake flasks with YP medium with 20 g L^-1^ d-fructose and 1 g L^-1^ d-glucose. Curves are shown for the HXT1p-GFP biosensor strains, and similar patterns were exhibited for the SUC2p-GFP and TPS1p-GFP biosensor strains as well.

There was no discernible difference in growth pattern between strains carrying the *HXT1*p-GFP biosensor and those carrying the *SUC2*p-GFP or *TPS1*p-GFP biosensor, neither in the background nor in the *pgi1*Δ genotype. This confirmed that the chosen biosensor system was non-invasive.

**S2– LC-MS analysis**

**Table S2.** Multiple-reaction-monitoring (MRM) transitions and retention times of LC-MS analysis of sugar phosphates (Rende et al. 2019). Relative standard deviations (RSD) were obtained as described in Materials and Methods. The internal standard (IS) used was 2-deoxy-d-glucose-6-phosphate.

| Compound | RT (min) | Quantifier | CE | Qualifier | CE | Method RSD  (%) |
| --- | --- | --- | --- | --- | --- | --- |
| 3-phosphoglyceric acid | 0.7 | 241→79 | 50 | 241→151 | 5 | 7.51 |
| Glyceraldehyde 3-P | 3.2 | 254→79 | 50 | 254→180 | 5 | 10.84 |
| Dihydroxyacetone phosphate | 2.9 | 254→79 | 50 | 254→180 | 5 | 22.22 |
| Fructose-1,6-PP | 4.2 | 536→79 | 50 | 536→159 | 21 | 22.18 |
| Erythrose-4-P | 5.3 | 340→79 | 50 | 340→266 | 5 | 25.58 |
| Xylulose-5-P | 7.4 | 426→79 | 49 | 426→37 | 9 | 13.77 (Sugars 5-P)  8.58 (Ribose-5-P) |
| Ribulose-5-P | 7.7 | 426→79 | 49 | 426→37 | 9 |  |
| Ribose-5-P | 8.7 | 426→79 | 49 | 426→37 | 9 |  |
| 2-Deoxyglucose 6-P (IS) | 7.9 | 440→79 | 41 | - | - | 12.03 |
| Galactose-1-P | 9.1 | 483→79 | 37 | 483→427 | 13 | 13.78 |
| Glucose-1-P | 9.9 | 483→79 | 37 | 483→427 | 13 | 13.94 |
| Fructose-6-P | 11.6 | 512→79 | 37 | 512→153 | 13 | 15.42 |
| Glucose-6-P | 11.7 | 512→79 | 37 | 512→153 | 13 | 15.05 |
| Seduheptulose-7-P | 12.6 | 598→79 | 49 | 598→542 | 17 | 21.82 |
| UDP-glucose/ UDP-galactose | 12.5 | 901→497 | 41 | 901→545 | 37 | 7.84 |
| Trehalose-6-P | 13.3 | 813→79 | 50 | 813→757 | 25 | 8.91 |
| 6-Phosphogluconate | 6.9 | 499→79 | 37 | - | - | 8.99 |

RT: Retention time, CE: Collision energy, RSD: Relative standard deviation.


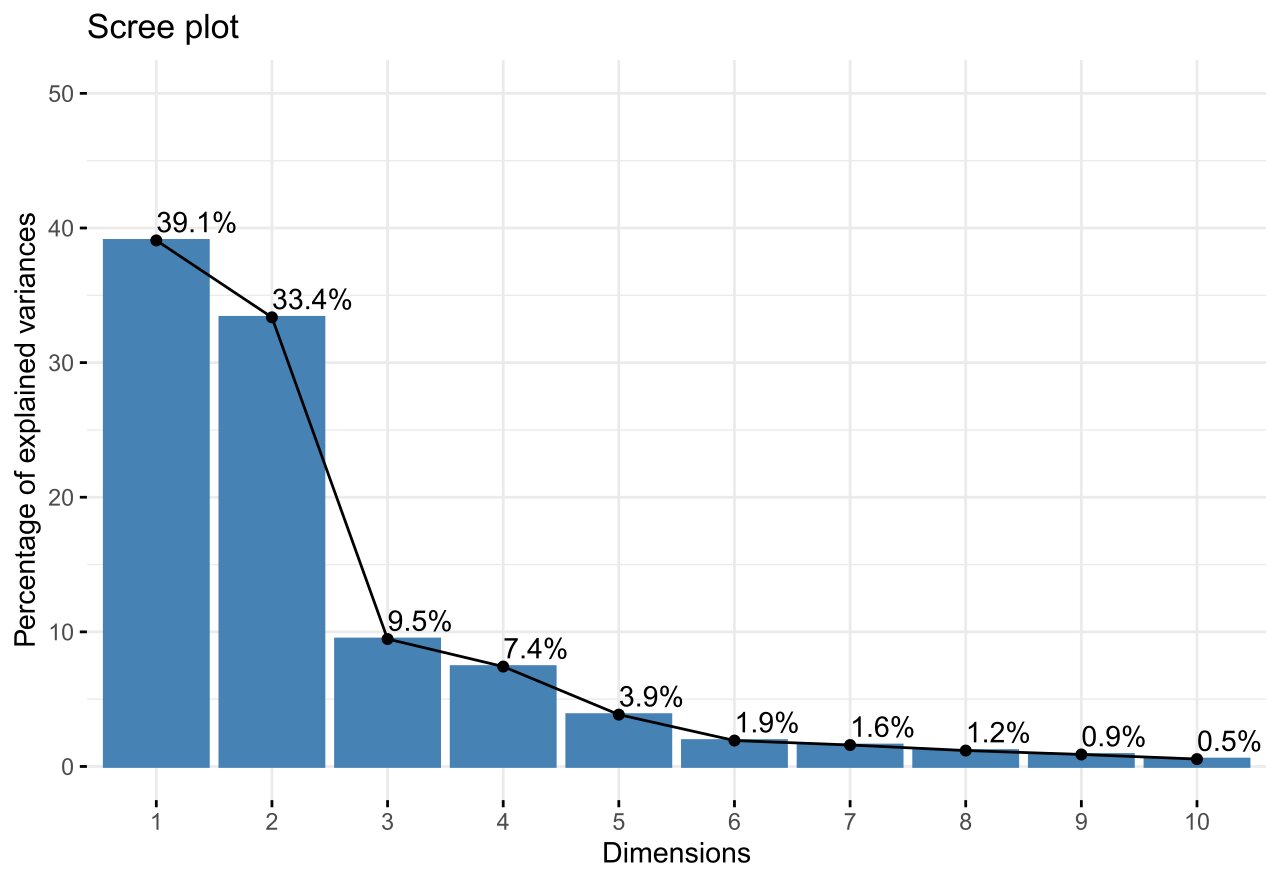


Figure S3. Top 10 principal components in metabolomics analysis explaining 99.4 % of the variation observed. There is a clear distinction between first two components and subsequent components.


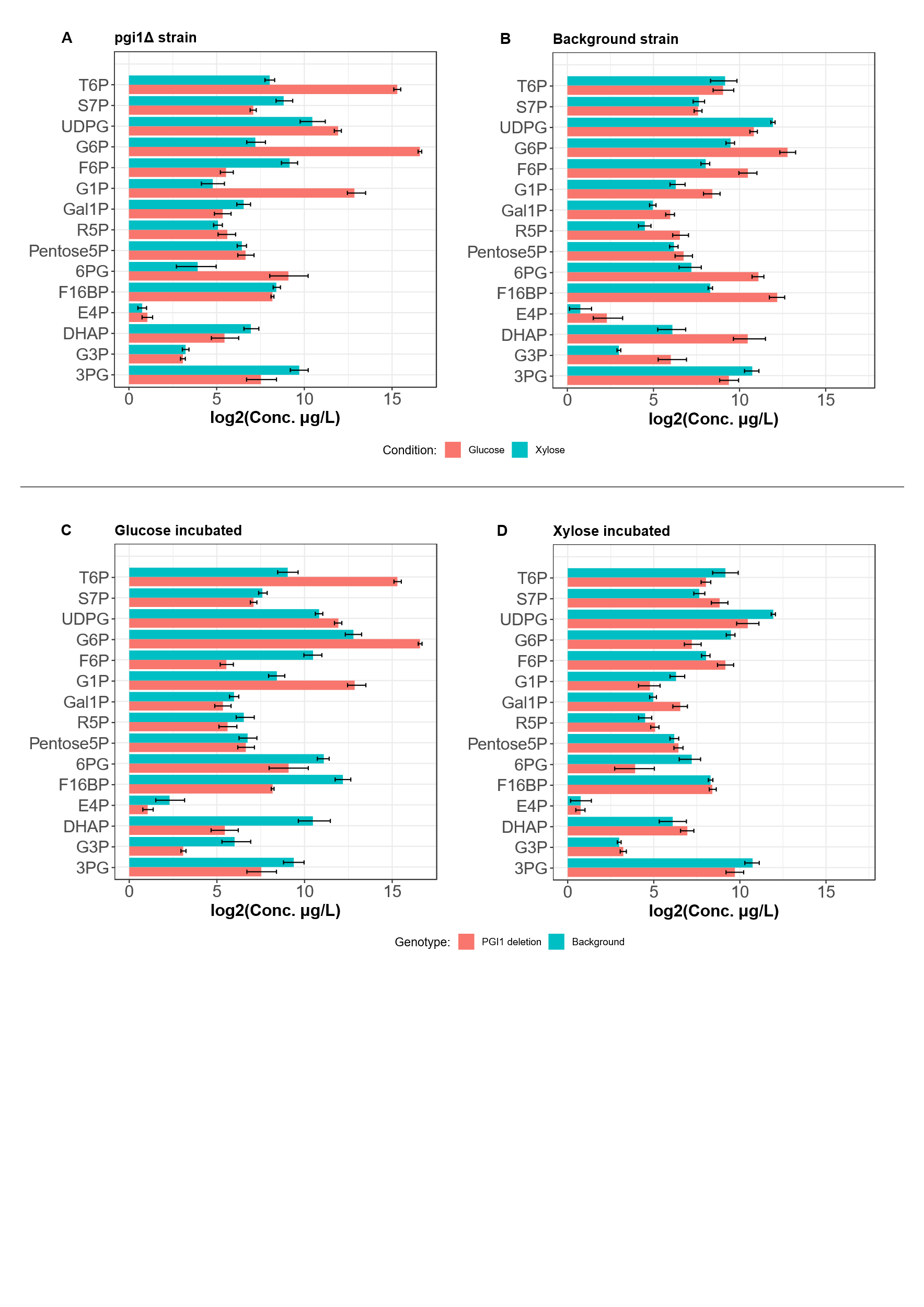


Figure S4. Concentration of intracellular sugar phosphates in background and pgi1Δ strains on 20 g L^-1^ d-glucose or 20 g L^-1^ d-xylose.

**S3 – Flow cytometry supplementary data**


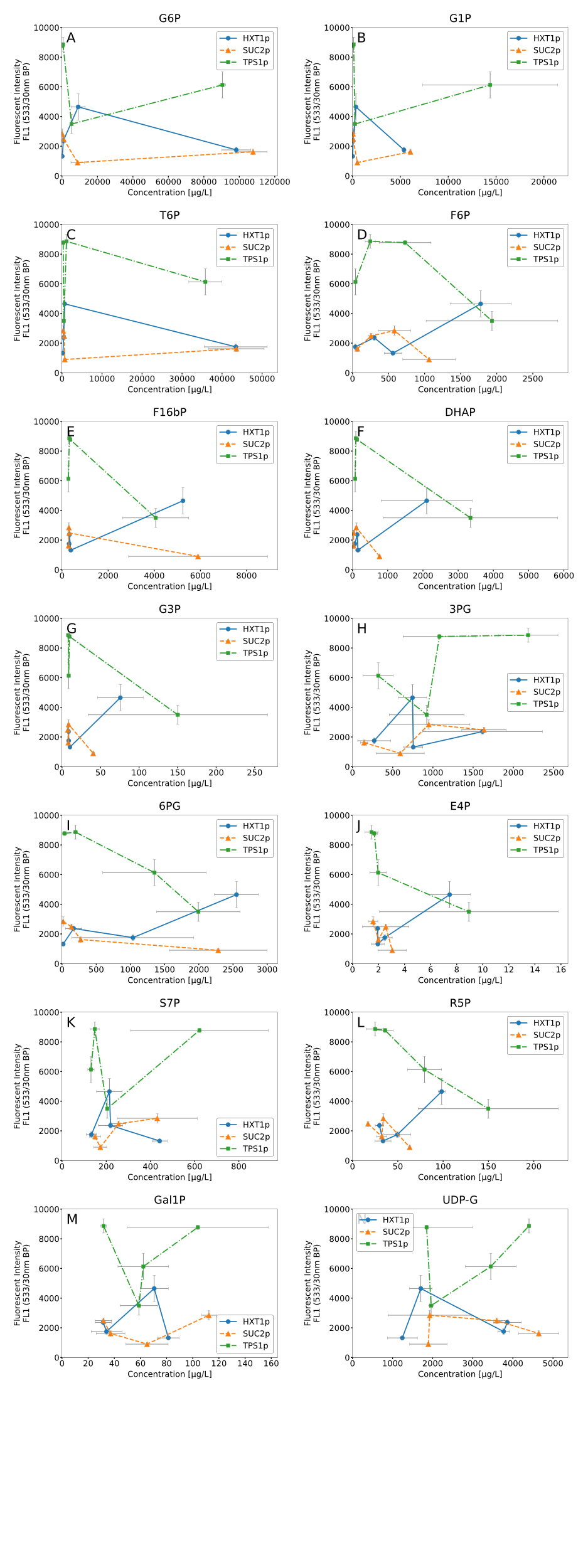


.

.

.


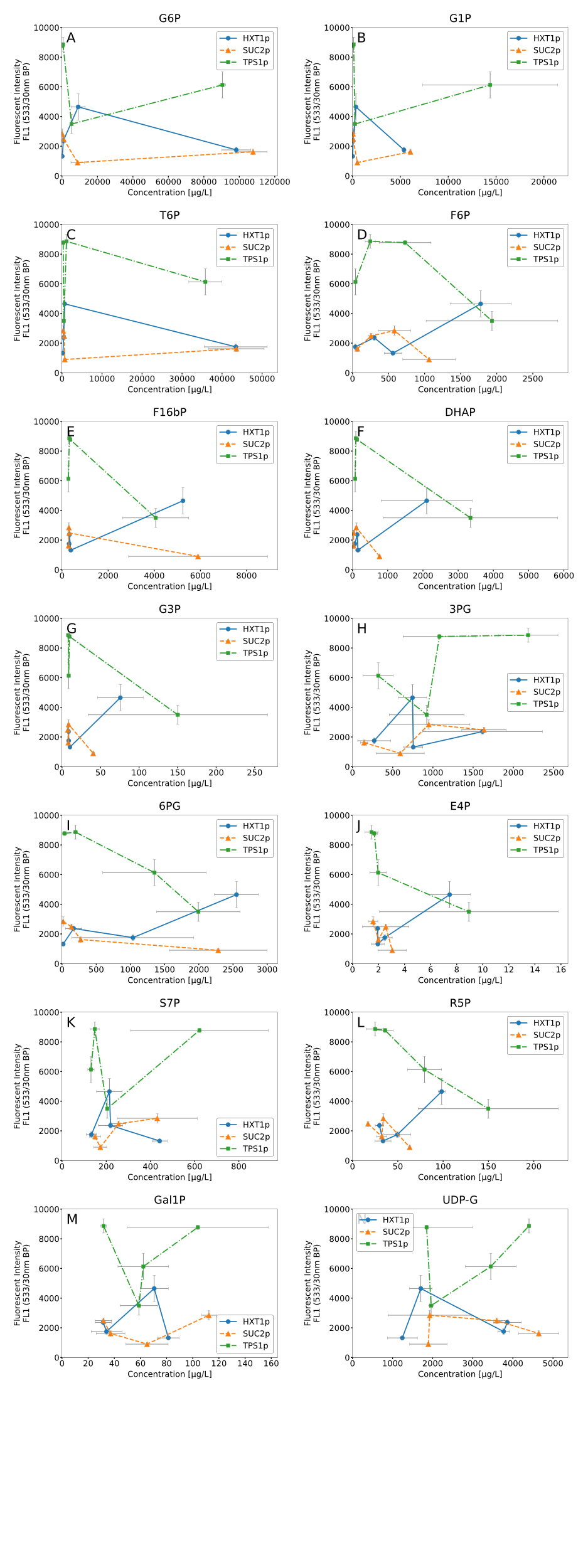


Figure S5. Fluorescence intensity (FI) of biosensors as a function of intracellular sugar phosphate concentrations obtained from the background and deletion strains grown on d-glucose or d-xylose. The biosensor for HXT1p (circles, blue solid line) reports on the Rgt2p/Snf3p pathway, SUC2p (triangles, orange dashed lines) the SNF1/Mig1p pathway and TPS1p (squares, green dot-dashed lines) the cAMP/PKA pathway. A: d-glucose-6-phosphate; B: d-glucose-1-phosphate; C: trehalose-6-phosphate; D: d-fructose-6-phosphate; E: d-fructose-1,6-bisphosphate; F: dihydroxyacetone; G: glyceraldehyde-3-phosphate; H: 3-phosphoglycerate; I: 6-phosphogluconate ; J: erythrose-4-phosphate ; K: sedoheptulose-7-phosphate; L: ribose-5-phosphate; M: d-galactose-1-phosphate; and N: uridine diphosphate hexose.

**Histograms from biosensors**

The fluorescence histograms for the biosensors with and without *PGI1* deletion are shown in Fig. S6. In the background strain (Fig. S6A, S6C & S6E) subpopulations with two fluorescence peaks are observed when cells are incubated with low levels of d-glucose and low levels of d-galactose. Subpopulations produced by the biosensor strains incubated on d-glucose have been observed previously (Brink et al., 2016; Osiro et al., 2018); in this case the heterogeneities are not severe enough to call for separate consideration, so they are treated as one population. These doubled peaks were not present in the *pgi1*Δ strains of the respective reporters on the same media (Fig. S6B, S6D & S6F).displayed more homogenous fluorescence populations than the background strains.


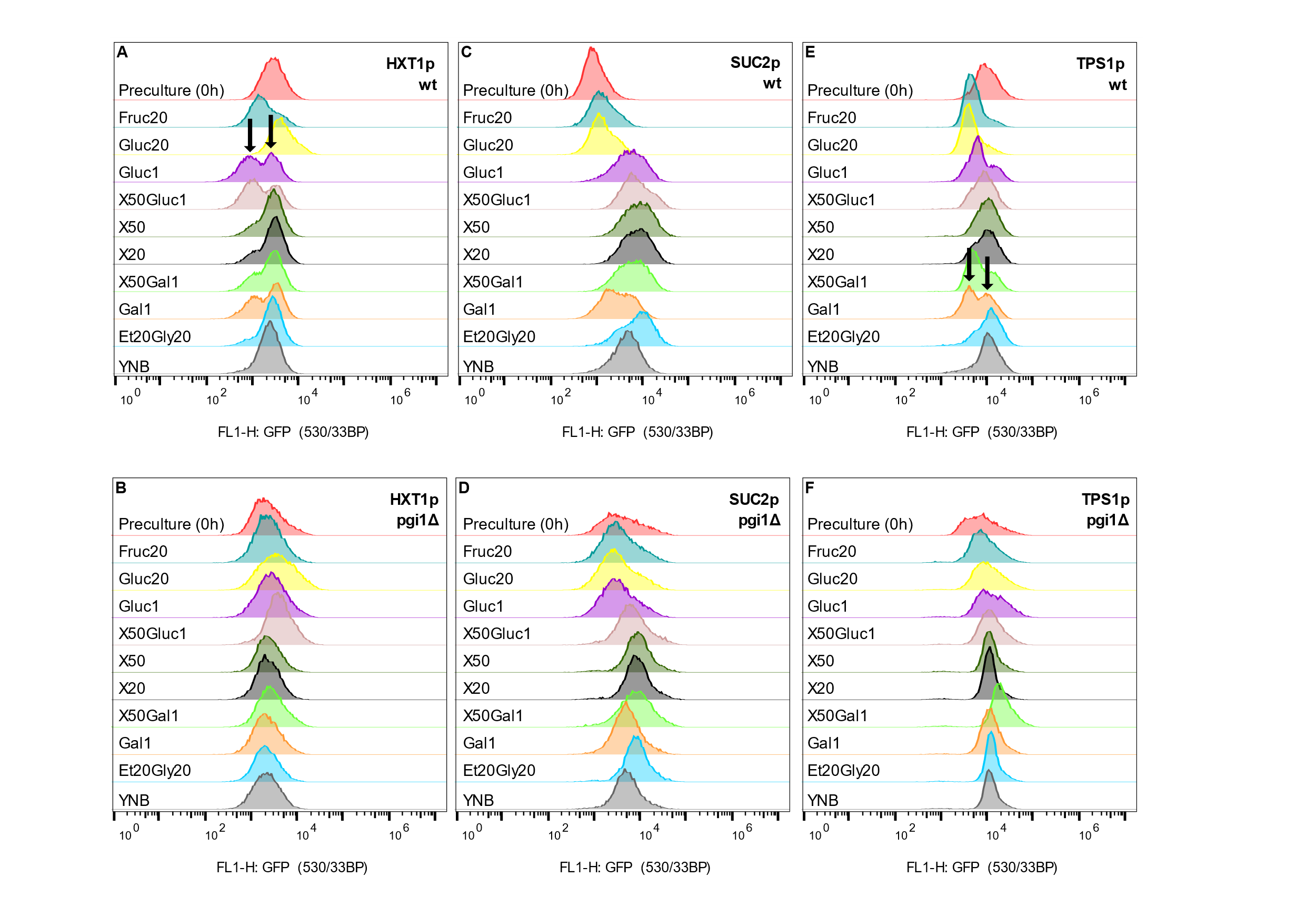


Figure S6. Histogram of biosensors HXT1p (A, B), SUC2p (C, D) and TPS1p (E,F) without (A, C, E) and with (B, D, F) PGI1 gene deletion. The four strains were analyzed at 0 h (=pre-culture conditions), and after 6 h for the following conditions: no carbon; 50 g L^-1^ d-xylose (X50); 1 g L^-1^ d-glucose (Gluc1); 50 g L^-1^ d-xylose and 1 g L^-1^ d-glucose (X50Gluc1); 1 g L^-1^ d-galactose (Gal1); 50 g L^-1^ d-xylose and 1 g L^-1^ d-galactose (X50Gal1); 20 g L^-1^ d-glucose (Gluc20); 20 g L^-1^ d-galactose (Gal20); and 20 g L^-1^ d-fructose (Fruc20). Black arrows indicate examples of subpopulations in fluorescence intensity.

**References**

Aguilera, A. (1986). *"Deletion of the phosphoglucose isomerase structural gene makes growth and sporulation glucose dependent in* Saccharomyces cerevisiae*."* Molecular and General Genetics 204: 310-316.

Brink, D. P., et al. (2016). *"Real-time monitoring of the sugar sensing in* Saccharomyces cerevisiae *indicates endogenous mechanisms for xylose signaling."* Microbial Cell Factories 15(1): 183.

Osiro, K. O., et al. (2018). *"Assessing the eff7ect of d-xylose on the sugar signaling pathways of* Saccharomyces cerevisiae *in strains engineered for xylose transport and assimilation."* FEMS Yeast Res 18(1).

Rende, U., et al. (2019). "*Two-step derivatization for determination of sugar phosphates in plants by combined reversed phase chromatography/tandem mass spectrometry*." Plant Methods 15(1): 127.
